# Supplementary figures and images for: The substrate-binding domains of the osmoregulatory ABC importer OpuA transiently interact
Source: eLife. 2024 May 2;12:RP90996. doi: 10.7554/eLife.90996 (PMC11065425; doi:10.7554/eLife.90996)

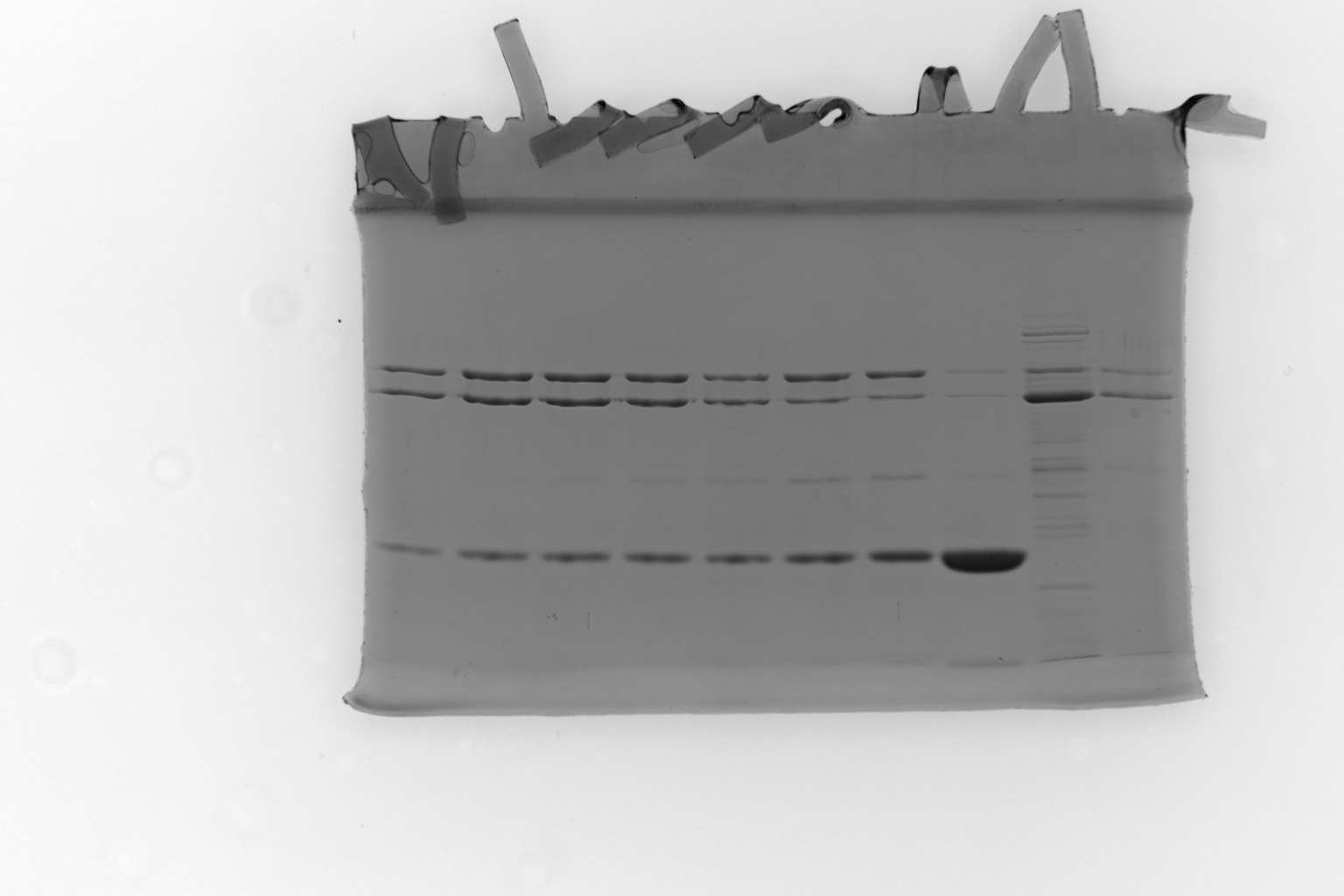

Supplement: Figure 1—source data 1. [file elife-90996-fig1-data1.zip › Figure 1C-Raw.png]

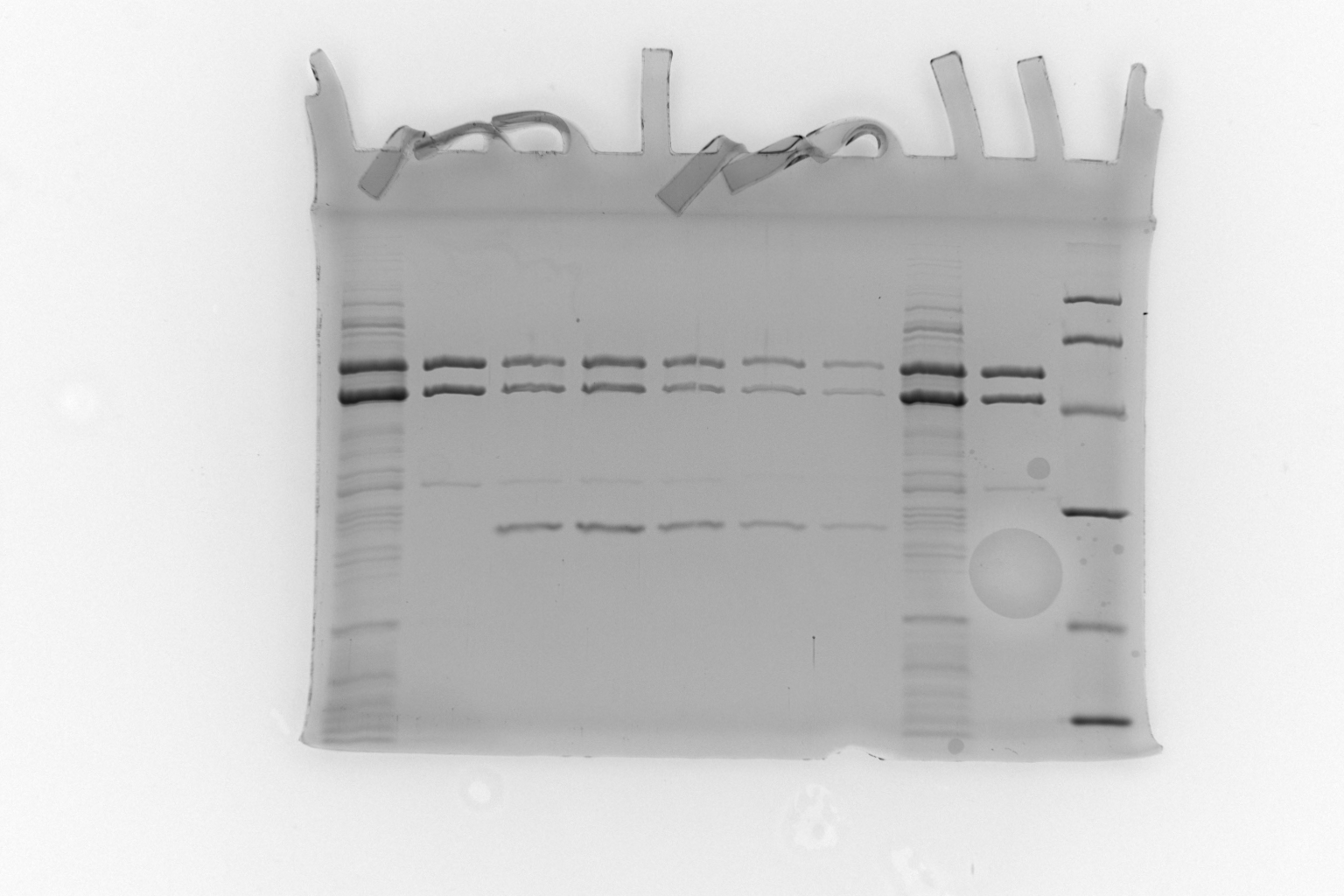

Supplement: Figure 1—source data 1. [file elife-90996-fig1-data1.zip › Figure 1D-Raw.png]

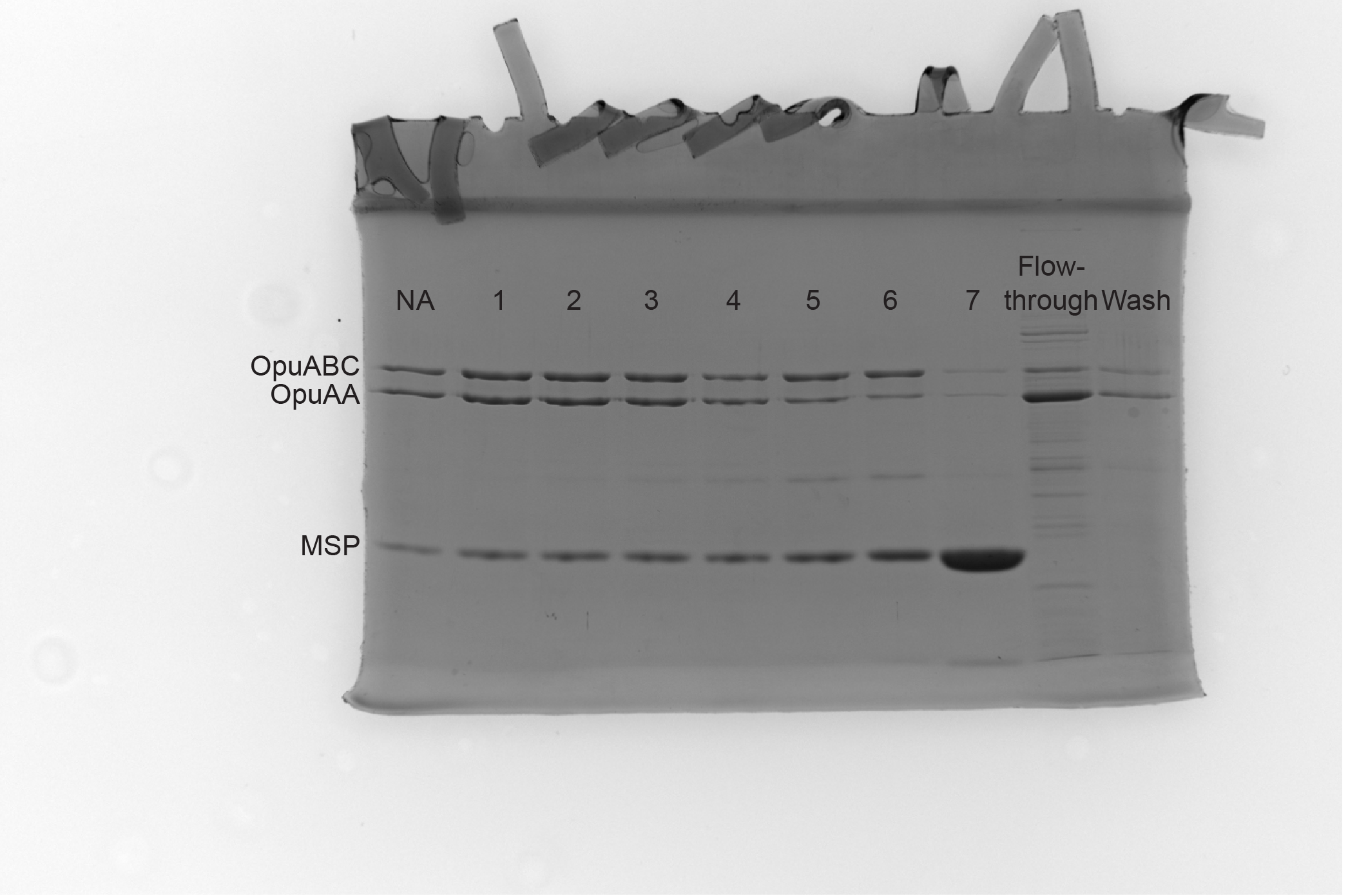

Supplement: Figure 1—source data 2. [file elife-90996-fig1-data2.zip › Figure 1C-RawLabelled.png]

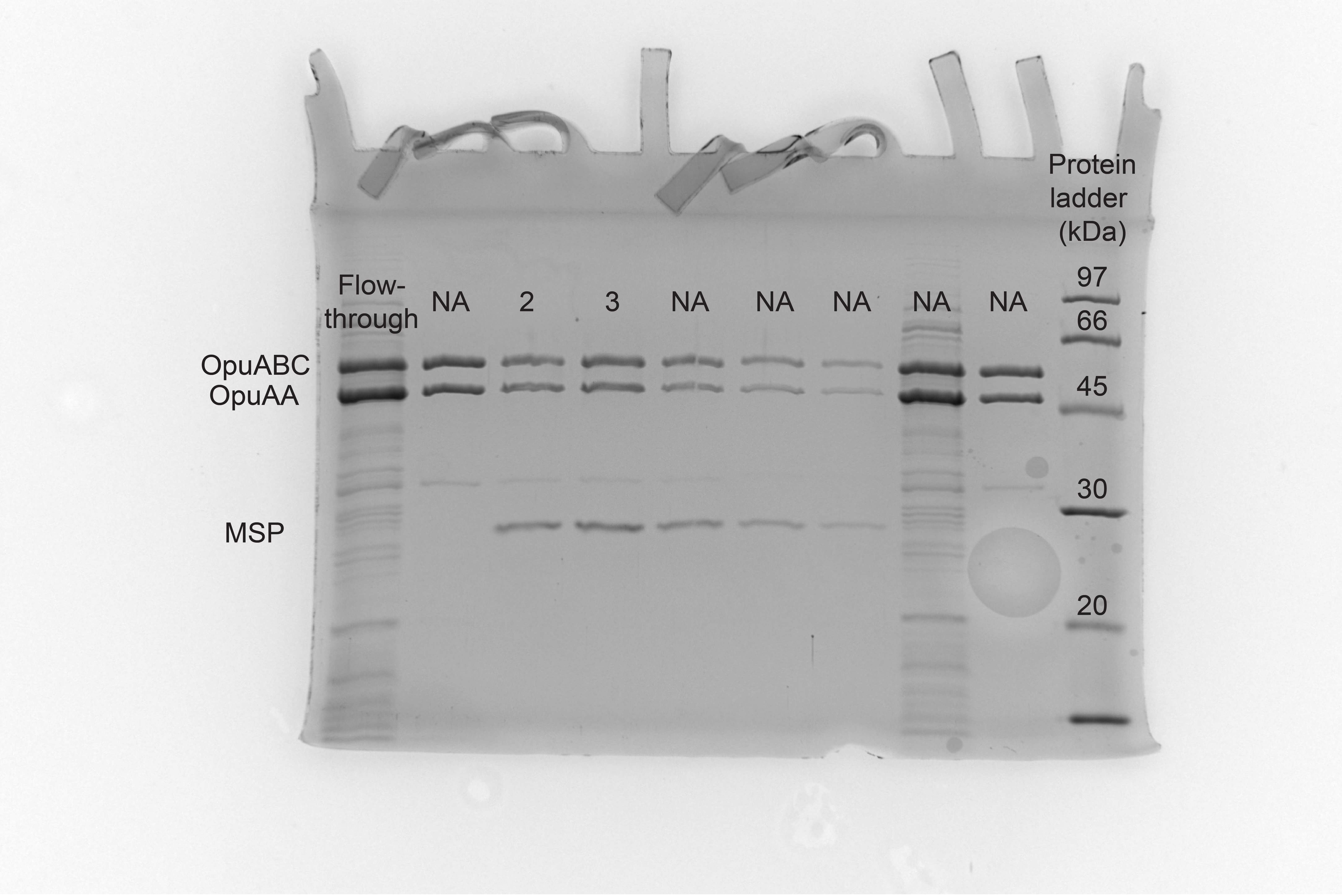

Supplement: Figure 1—source data 2. [file elife-90996-fig1-data2.zip › Figure 1D-RawLabelled.png]

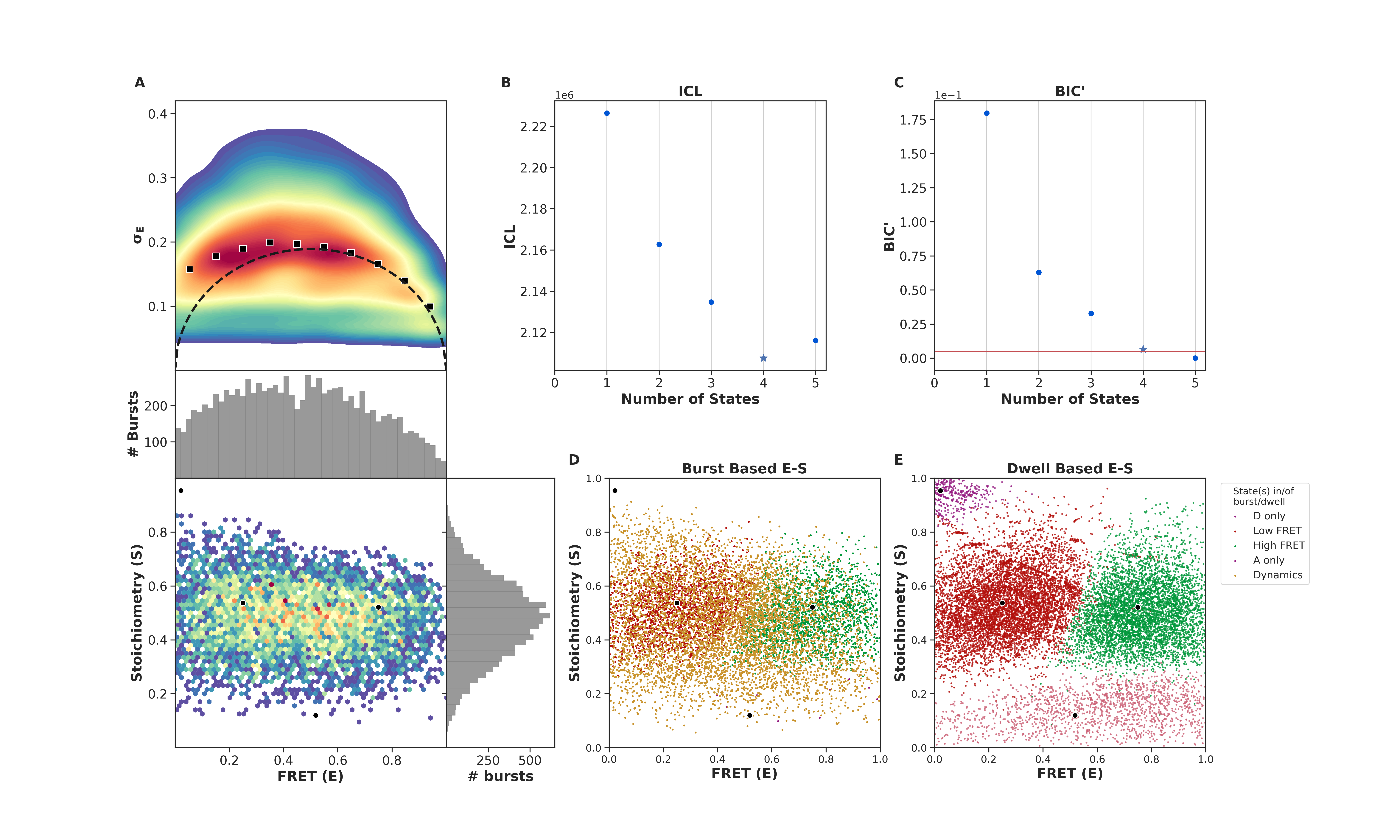

Supplement: Table 2—source data 1. — (A) From top to bottom: (1) Burst variance analysis of the bursts which were corrected by the leakage, crosstalk, and γ-correction factors and which were selected after removing donor-only and acceptor-only bursts. The standard deviation of FRET in each burst is plotted against its mean FRET. Black squares show average values per FRET bin. Black dotted line shows the expected standard deviation in the absence of within-burst dynamics. (2) 2D E-S histogram shows the same data as in (1), with on both sides a histogram that represents the same bursts. (B) Plot of the ICL-values for each final model. The model used in the downstream analysis and following figures is shown as a star. (C) Plot of the BIC’-values for each final model. The red line represents a 0.05 cut-off. The model used is shown as a star. (D) Burst-based 2D E-S scatter plot. Bursts are colored on the basis of the assigned state of the chosen mpH2MM model. If a burst contains more than one state, it is assigned as being dynamic. (E) Dwell-based 2D E-S scatter plot. Dwells are colored on the basis of the assigned state of the chosen mpH2MM model. The dwells were corrected for leakage, direct excitation and the γ-factor. Black dots in A, D and E represent the average value of each state. [file elife-90996-table2-data1.zip › Table 2-Source Data 1/50BisTris0KCl.png]

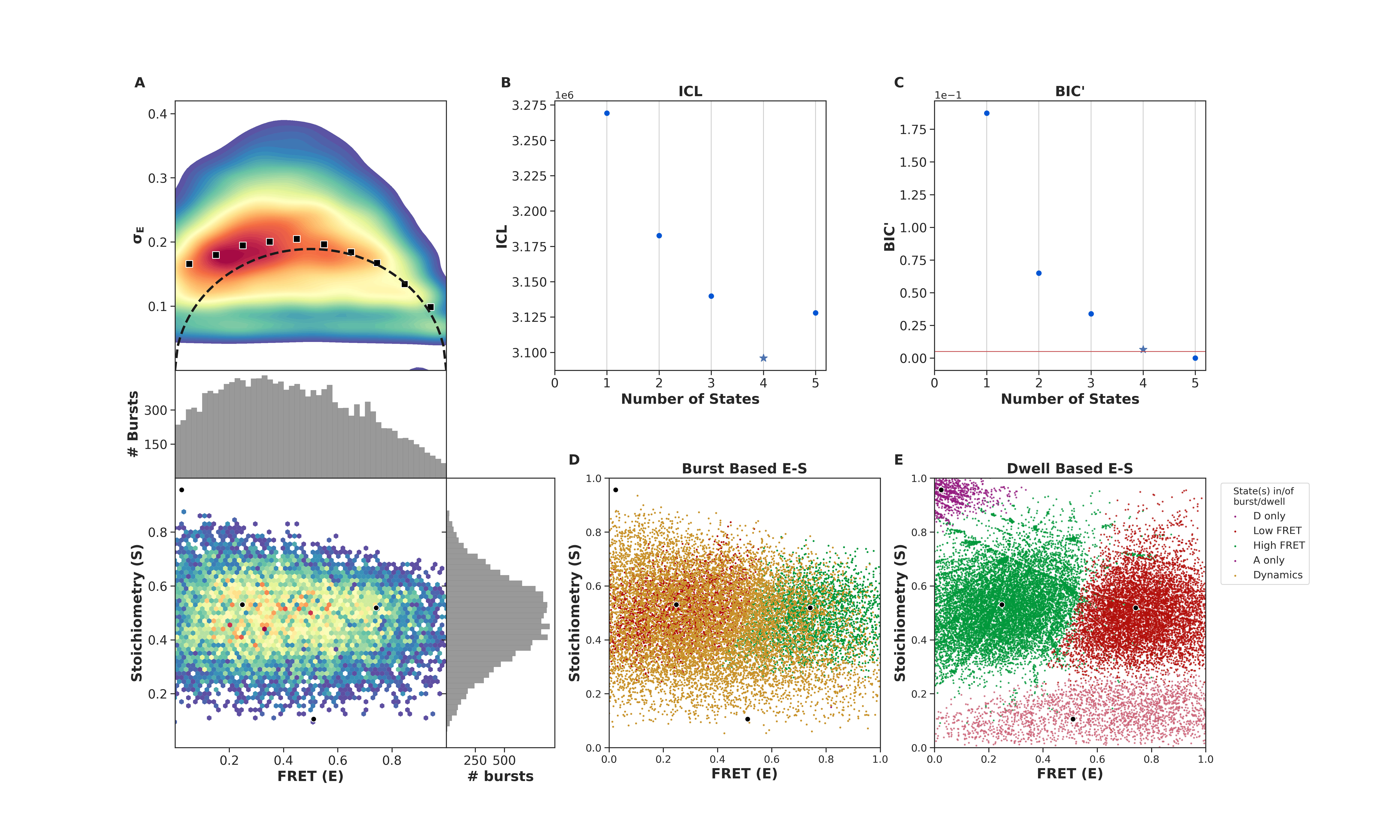

Supplement: Table 2—source data 1. — (A) From top to bottom: (1) Burst variance analysis of the bursts which were corrected by the leakage, crosstalk, and γ-correction factors and which were selected after removing donor-only and acceptor-only bursts. The standard deviation of FRET in each burst is plotted against its mean FRET. Black squares show average values per FRET bin. Black dotted line shows the expected standard deviation in the absence of within-burst dynamics. (2) 2D E-S histogram shows the same data as in (1), with on both sides a histogram that represents the same bursts. (B) Plot of the ICL-values for each final model. The model used in the downstream analysis and following figures is shown as a star. (C) Plot of the BIC’-values for each final model. The red line represents a 0.05 cut-off. The model used is shown as a star. (D) Burst-based 2D E-S scatter plot. Bursts are colored on the basis of the assigned state of the chosen mpH2MM model. If a burst contains more than one state, it is assigned as being dynamic. (E) Dwell-based 2D E-S scatter plot. Dwells are colored on the basis of the assigned state of the chosen mpH2MM model. The dwells were corrected for leakage, direct excitation and the γ-factor. Black dots in A, D and E represent the average value of each state. [file elife-90996-table2-data1.zip › Table 2-Source Data 1/50HEPES0KCl.png]

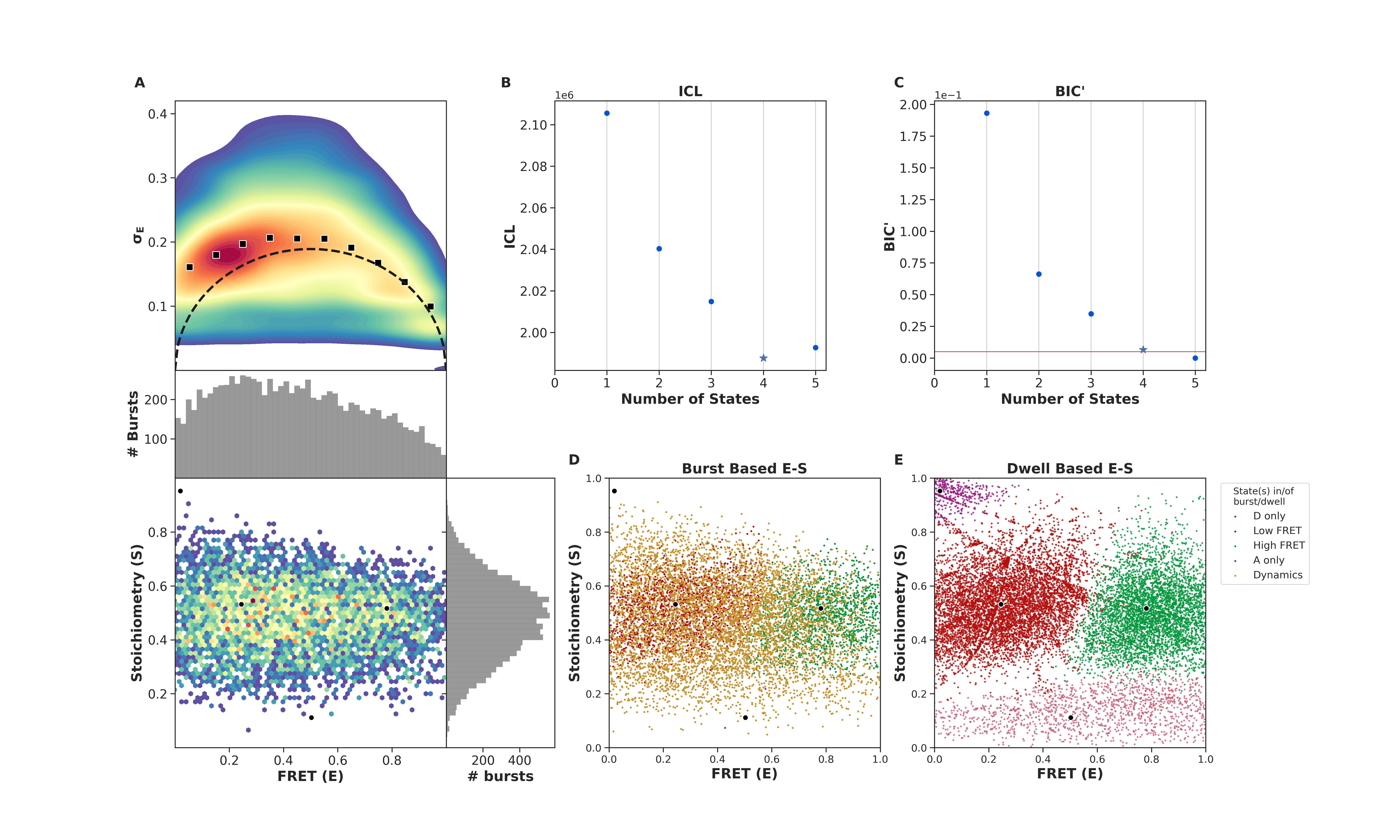

Supplement: Table 2—source data 1. — (A) From top to bottom: (1) Burst variance analysis of the bursts which were corrected by the leakage, crosstalk, and γ-correction factors and which were selected after removing donor-only and acceptor-only bursts. The standard deviation of FRET in each burst is plotted against its mean FRET. Black squares show average values per FRET bin. Black dotted line shows the expected standard deviation in the absence of within-burst dynamics. (2) 2D E-S histogram shows the same data as in (1), with on both sides a histogram that represents the same bursts. (B) Plot of the ICL-values for each final model. The model used in the downstream analysis and following figures is shown as a star. (C) Plot of the BIC’-values for each final model. The red line represents a 0.05 cut-off. The model used is shown as a star. (D) Burst-based 2D E-S scatter plot. Bursts are colored on the basis of the assigned state of the chosen mpH2MM model. If a burst contains more than one state, it is assigned as being dynamic. (E) Dwell-based 2D E-S scatter plot. Dwells are colored on the basis of the assigned state of the chosen mpH2MM model. The dwells were corrected for leakage, direct excitation and the γ-factor. Black dots in A, D and E represent the average value of each state. [file elife-90996-table2-data1.zip › Table 2-Source Data 1/50HEPES0KCl100GB.png]

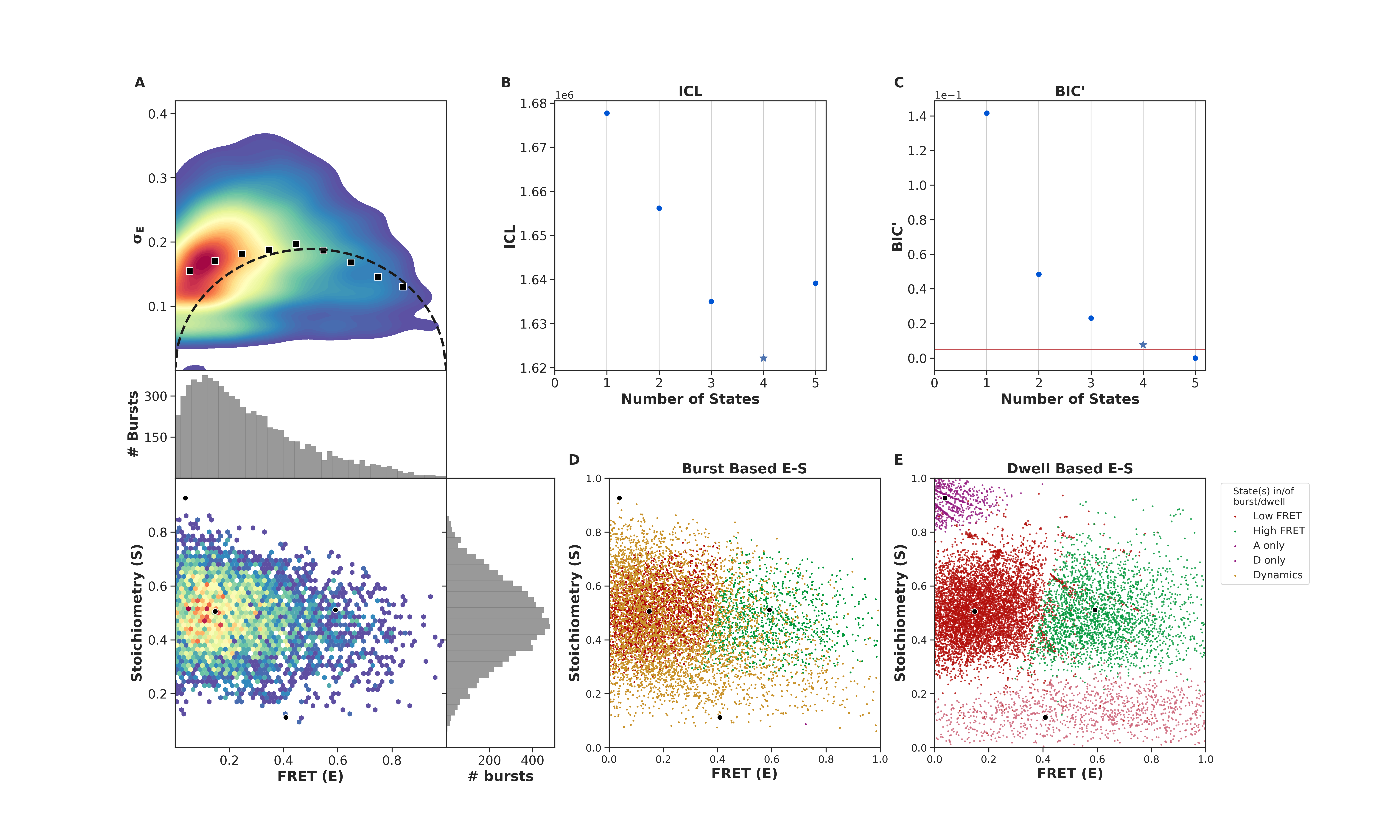

Supplement: Table 2—source data 1. — (A) From top to bottom: (1) Burst variance analysis of the bursts which were corrected by the leakage, crosstalk, and γ-correction factors and which were selected after removing donor-only and acceptor-only bursts. The standard deviation of FRET in each burst is plotted against its mean FRET. Black squares show average values per FRET bin. Black dotted line shows the expected standard deviation in the absence of within-burst dynamics. (2) 2D E-S histogram shows the same data as in (1), with on both sides a histogram that represents the same bursts. (B) Plot of the ICL-values for each final model. The model used in the downstream analysis and following figures is shown as a star. (C) Plot of the BIC’-values for each final model. The red line represents a 0.05 cut-off. The model used is shown as a star. (D) Burst-based 2D E-S scatter plot. Bursts are colored on the basis of the assigned state of the chosen mpH2MM model. If a burst contains more than one state, it is assigned as being dynamic. (E) Dwell-based 2D E-S scatter plot. Dwells are colored on the basis of the assigned state of the chosen mpH2MM model. The dwells were corrected for leakage, direct excitation and the γ-factor. Black dots in A, D and E represent the average value of each state. [file elife-90996-table2-data1.zip › Table 2-Source Data 1/50HEPES1000KCl.png]

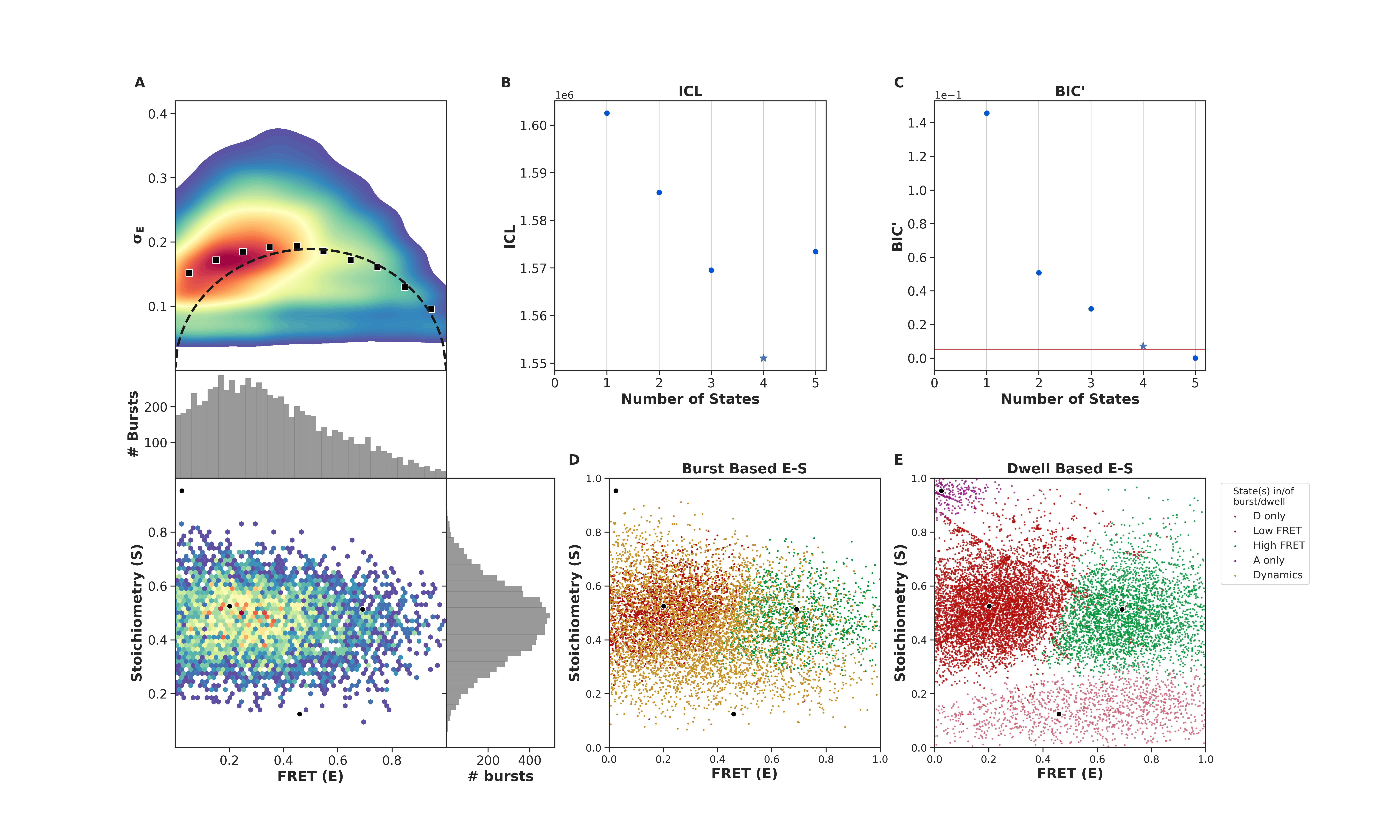

Supplement: Table 2—source data 1. — (A) From top to bottom: (1) Burst variance analysis of the bursts which were corrected by the leakage, crosstalk, and γ-correction factors and which were selected after removing donor-only and acceptor-only bursts. The standard deviation of FRET in each burst is plotted against its mean FRET. Black squares show average values per FRET bin. Black dotted line shows the expected standard deviation in the absence of within-burst dynamics. (2) 2D E-S histogram shows the same data as in (1), with on both sides a histogram that represents the same bursts. (B) Plot of the ICL-values for each final model. The model used in the downstream analysis and following figures is shown as a star. (C) Plot of the BIC’-values for each final model. The red line represents a 0.05 cut-off. The model used is shown as a star. (D) Burst-based 2D E-S scatter plot. Bursts are colored on the basis of the assigned state of the chosen mpH2MM model. If a burst contains more than one state, it is assigned as being dynamic. (E) Dwell-based 2D E-S scatter plot. Dwells are colored on the basis of the assigned state of the chosen mpH2MM model. The dwells were corrected for leakage, direct excitation and the γ-factor. Black dots in A, D and E represent the average value of each state. [file elife-90996-table2-data1.zip › Table 2-Source Data 1/50HEPES100KCl.png]

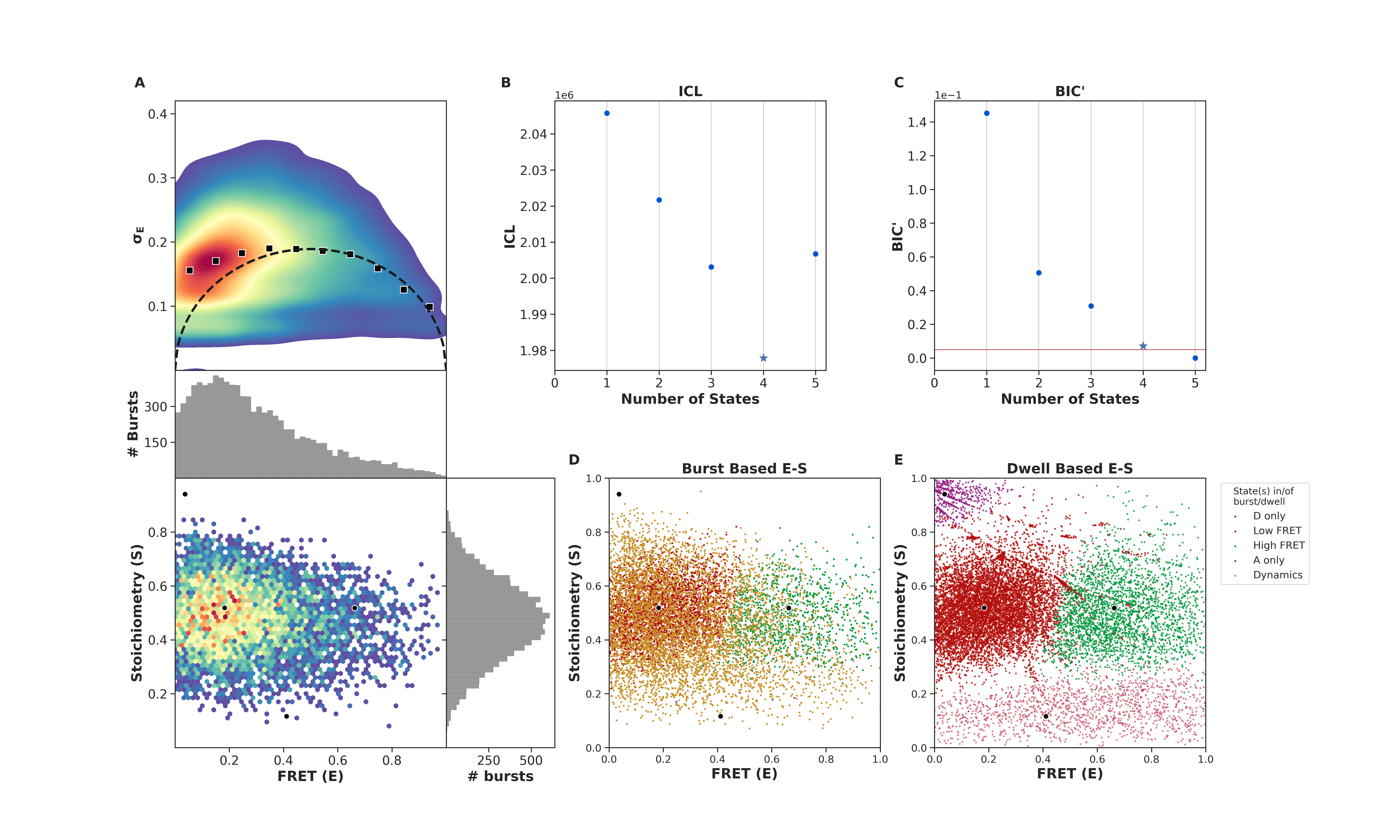

Supplement: Table 2—source data 1. — (A) From top to bottom: (1) Burst variance analysis of the bursts which were corrected by the leakage, crosstalk, and γ-correction factors and which were selected after removing donor-only and acceptor-only bursts. The standard deviation of FRET in each burst is plotted against its mean FRET. Black squares show average values per FRET bin. Black dotted line shows the expected standard deviation in the absence of within-burst dynamics. (2) 2D E-S histogram shows the same data as in (1), with on both sides a histogram that represents the same bursts. (B) Plot of the ICL-values for each final model. The model used in the downstream analysis and following figures is shown as a star. (C) Plot of the BIC’-values for each final model. The red line represents a 0.05 cut-off. The model used is shown as a star. (D) Burst-based 2D E-S scatter plot. Bursts are colored on the basis of the assigned state of the chosen mpH2MM model. If a burst contains more than one state, it is assigned as being dynamic. (E) Dwell-based 2D E-S scatter plot. Dwells are colored on the basis of the assigned state of the chosen mpH2MM model. The dwells were corrected for leakage, direct excitation and the γ-factor. Black dots in A, D and E represent the average value of each state. [file elife-90996-table2-data1.zip › Table 2-Source Data 1/50HEPES200KCl.png]

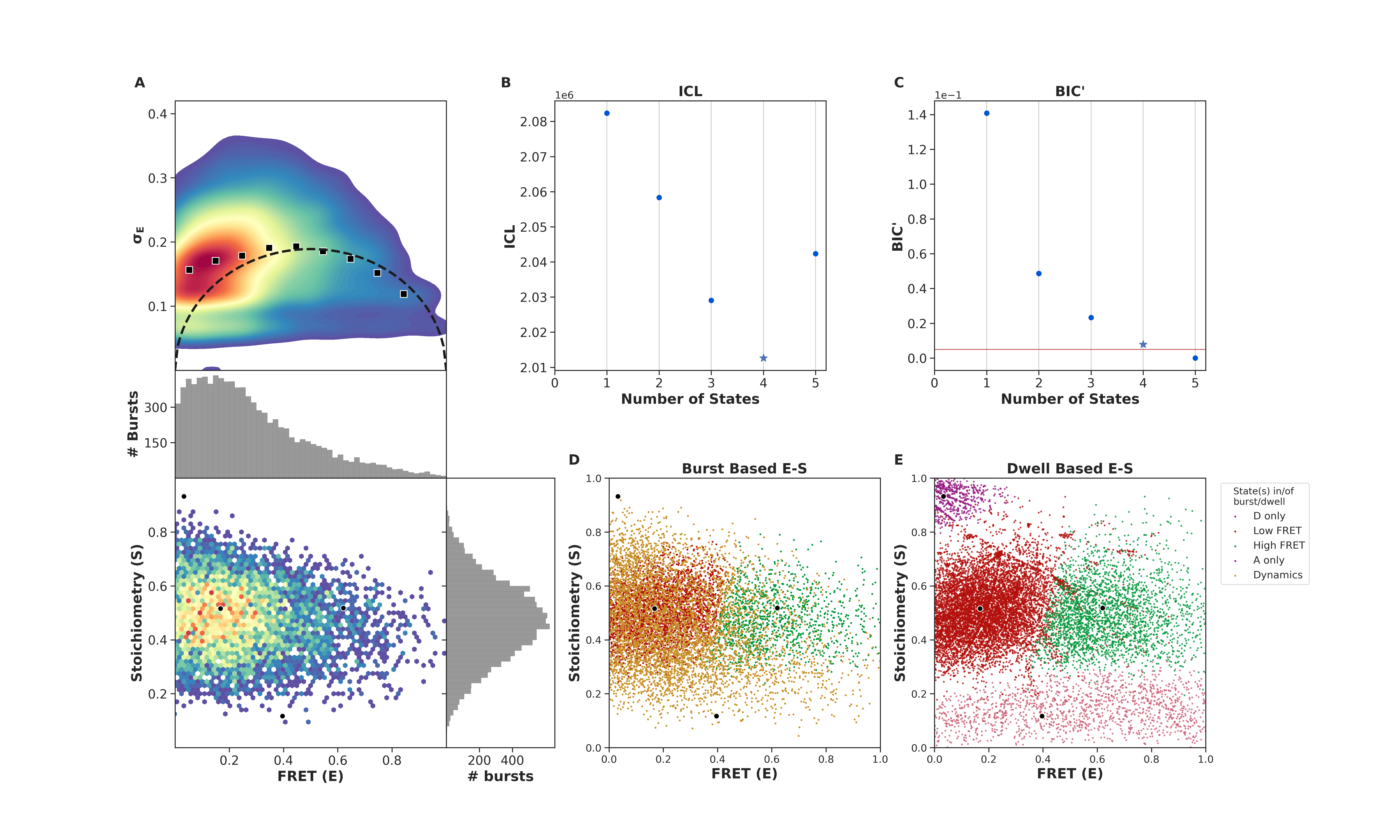

Supplement: Table 2—source data 1. — (A) From top to bottom: (1) Burst variance analysis of the bursts which were corrected by the leakage, crosstalk, and γ-correction factors and which were selected after removing donor-only and acceptor-only bursts. The standard deviation of FRET in each burst is plotted against its mean FRET. Black squares show average values per FRET bin. Black dotted line shows the expected standard deviation in the absence of within-burst dynamics. (2) 2D E-S histogram shows the same data as in (1), with on both sides a histogram that represents the same bursts. (B) Plot of the ICL-values for each final model. The model used in the downstream analysis and following figures is shown as a star. (C) Plot of the BIC’-values for each final model. The red line represents a 0.05 cut-off. The model used is shown as a star. (D) Burst-based 2D E-S scatter plot. Bursts are colored on the basis of the assigned state of the chosen mpH2MM model. If a burst contains more than one state, it is assigned as being dynamic. (E) Dwell-based 2D E-S scatter plot. Dwells are colored on the basis of the assigned state of the chosen mpH2MM model. The dwells were corrected for leakage, direct excitation and the γ-factor. Black dots in A, D and E represent the average value of each state. [file elife-90996-table2-data1.zip › Table 2-Source Data 1/50HEPES400KCl.png]

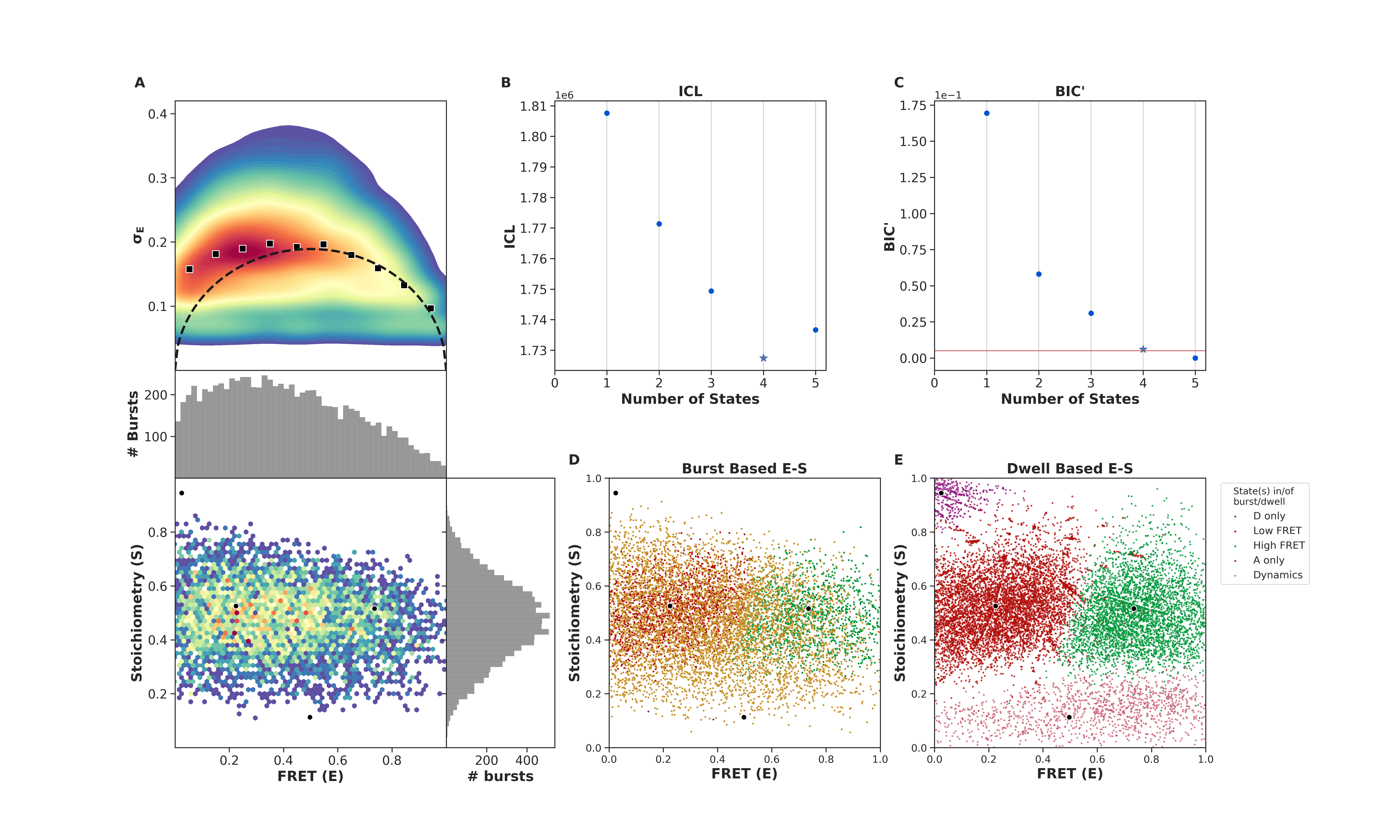

Supplement: Table 2—source data 1. — (A) From top to bottom: (1) Burst variance analysis of the bursts which were corrected by the leakage, crosstalk, and γ-correction factors and which were selected after removing donor-only and acceptor-only bursts. The standard deviation of FRET in each burst is plotted against its mean FRET. Black squares show average values per FRET bin. Black dotted line shows the expected standard deviation in the absence of within-burst dynamics. (2) 2D E-S histogram shows the same data as in (1), with on both sides a histogram that represents the same bursts. (B) Plot of the ICL-values for each final model. The model used in the downstream analysis and following figures is shown as a star. (C) Plot of the BIC’-values for each final model. The red line represents a 0.05 cut-off. The model used is shown as a star. (D) Burst-based 2D E-S scatter plot. Bursts are colored on the basis of the assigned state of the chosen mpH2MM model. If a burst contains more than one state, it is assigned as being dynamic. (E) Dwell-based 2D E-S scatter plot. Dwells are colored on the basis of the assigned state of the chosen mpH2MM model. The dwells were corrected for leakage, direct excitation and the γ-factor. Black dots in A, D and E represent the average value of each state. [file elife-90996-table2-data1.zip › Table 2-Source Data 1/50HEPES50KCl.png]

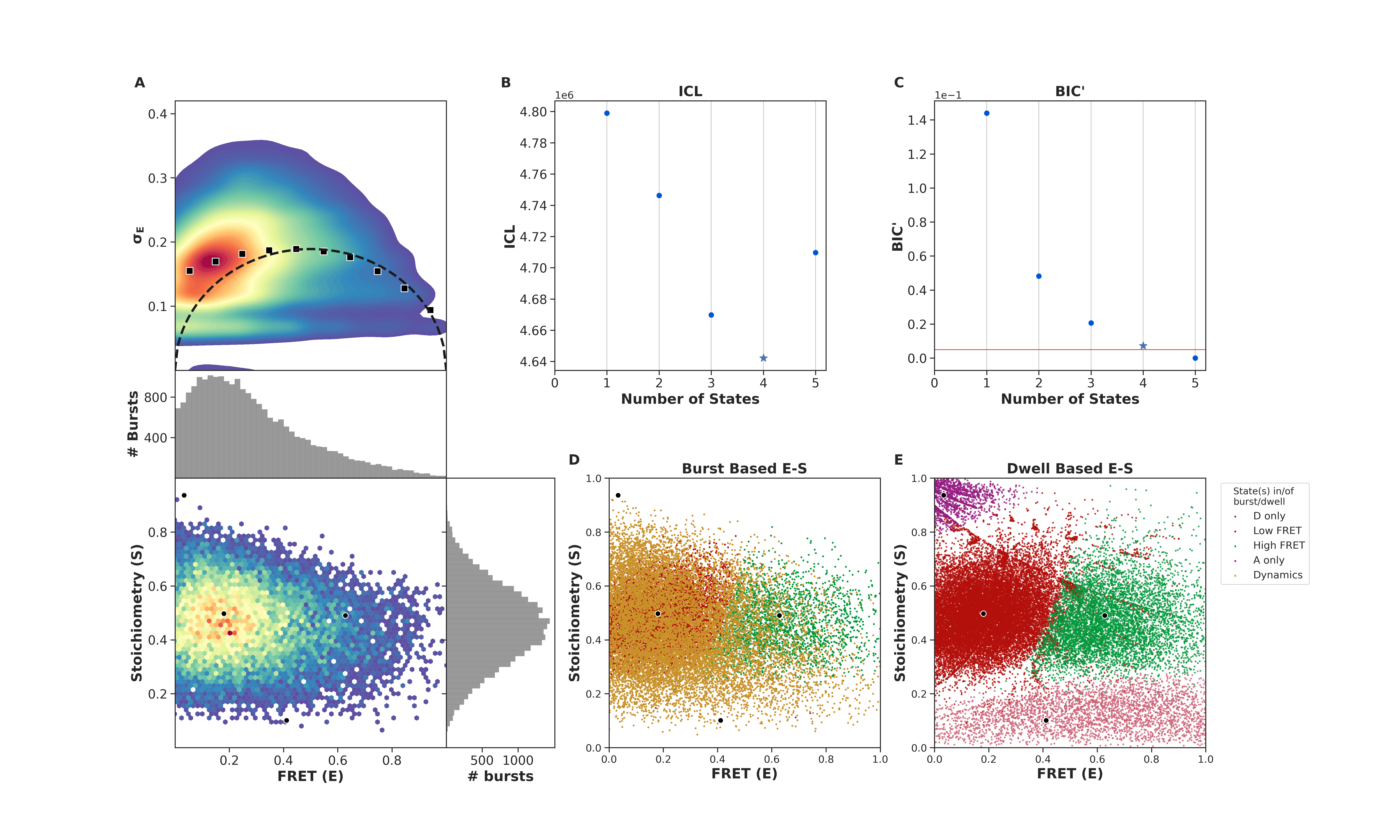

Supplement: Table 2—source data 1. — (A) From top to bottom: (1) Burst variance analysis of the bursts which were corrected by the leakage, crosstalk, and γ-correction factors and which were selected after removing donor-only and acceptor-only bursts. The standard deviation of FRET in each burst is plotted against its mean FRET. Black squares show average values per FRET bin. Black dotted line shows the expected standard deviation in the absence of within-burst dynamics. (2) 2D E-S histogram shows the same data as in (1), with on both sides a histogram that represents the same bursts. (B) Plot of the ICL-values for each final model. The model used in the downstream analysis and following figures is shown as a star. (C) Plot of the BIC’-values for each final model. The red line represents a 0.05 cut-off. The model used is shown as a star. (D) Burst-based 2D E-S scatter plot. Bursts are colored on the basis of the assigned state of the chosen mpH2MM model. If a burst contains more than one state, it is assigned as being dynamic. (E) Dwell-based 2D E-S scatter plot. Dwells are colored on the basis of the assigned state of the chosen mpH2MM model. The dwells were corrected for leakage, direct excitation and the γ-factor. Black dots in A, D and E represent the average value of each state. [file elife-90996-table2-data1.zip › Table 2-Source Data 1/50HEPES600KCl.png]

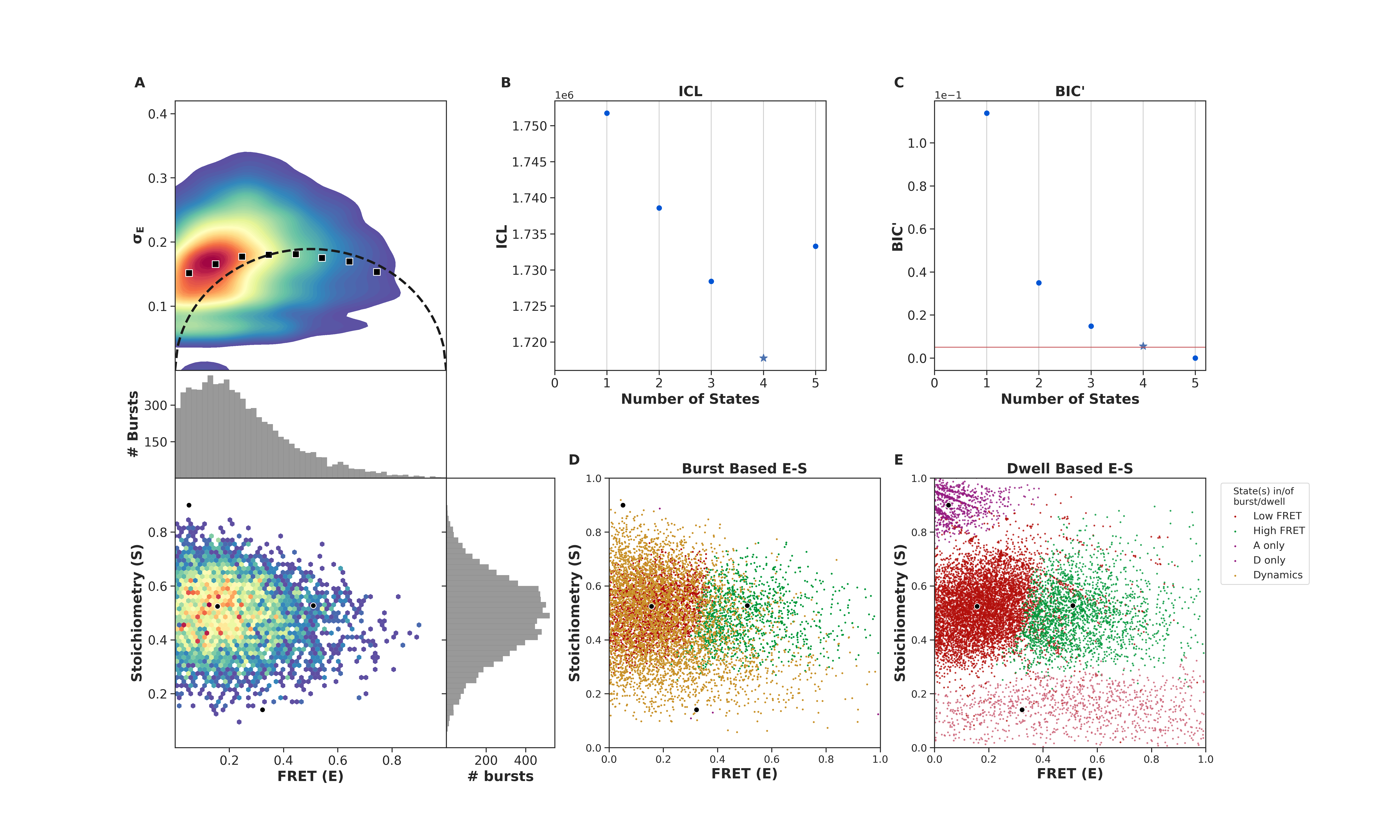

Supplement: Table 2—source data 1. — (A) From top to bottom: (1) Burst variance analysis of the bursts which were corrected by the leakage, crosstalk, and γ-correction factors and which were selected after removing donor-only and acceptor-only bursts. The standard deviation of FRET in each burst is plotted against its mean FRET. Black squares show average values per FRET bin. Black dotted line shows the expected standard deviation in the absence of within-burst dynamics. (2) 2D E-S histogram shows the same data as in (1), with on both sides a histogram that represents the same bursts. (B) Plot of the ICL-values for each final model. The model used in the downstream analysis and following figures is shown as a star. (C) Plot of the BIC’-values for each final model. The red line represents a 0.05 cut-off. The model used is shown as a star. (D) Burst-based 2D E-S scatter plot. Bursts are colored on the basis of the assigned state of the chosen mpH2MM model. If a burst contains more than one state, it is assigned as being dynamic. (E) Dwell-based 2D E-S scatter plot. Dwells are colored on the basis of the assigned state of the chosen mpH2MM model. The dwells were corrected for leakage, direct excitation and the γ-factor. Black dots in A, D and E represent the average value of each state. [file elife-90996-table2-data1.zip › Table 2-Source Data 1/50HEPES600KCl20MgATP.png]

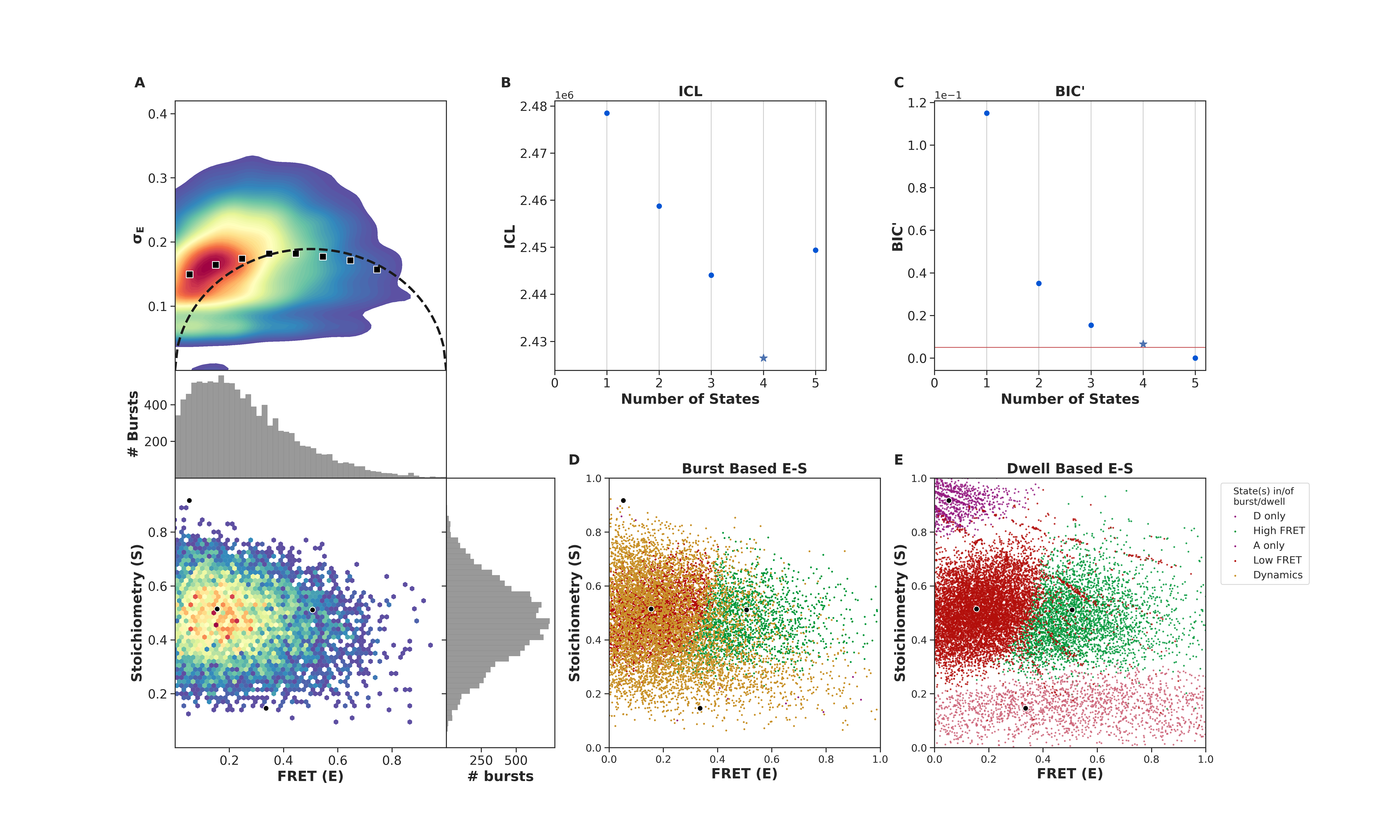

Supplement: Table 2—source data 1. — (A) From top to bottom: (1) Burst variance analysis of the bursts which were corrected by the leakage, crosstalk, and γ-correction factors and which were selected after removing donor-only and acceptor-only bursts. The standard deviation of FRET in each burst is plotted against its mean FRET. Black squares show average values per FRET bin. Black dotted line shows the expected standard deviation in the absence of within-burst dynamics. (2) 2D E-S histogram shows the same data as in (1), with on both sides a histogram that represents the same bursts. (B) Plot of the ICL-values for each final model. The model used in the downstream analysis and following figures is shown as a star. (C) Plot of the BIC’-values for each final model. The red line represents a 0.05 cut-off. The model used is shown as a star. (D) Burst-based 2D E-S scatter plot. Bursts are colored on the basis of the assigned state of the chosen mpH2MM model. If a burst contains more than one state, it is assigned as being dynamic. (E) Dwell-based 2D E-S scatter plot. Dwells are colored on the basis of the assigned state of the chosen mpH2MM model. The dwells were corrected for leakage, direct excitation and the γ-factor. Black dots in A, D and E represent the average value of each state. [file elife-90996-table2-data1.zip › Table 2-Source Data 1/50HEPES600KCl20MgATP100GB.png]

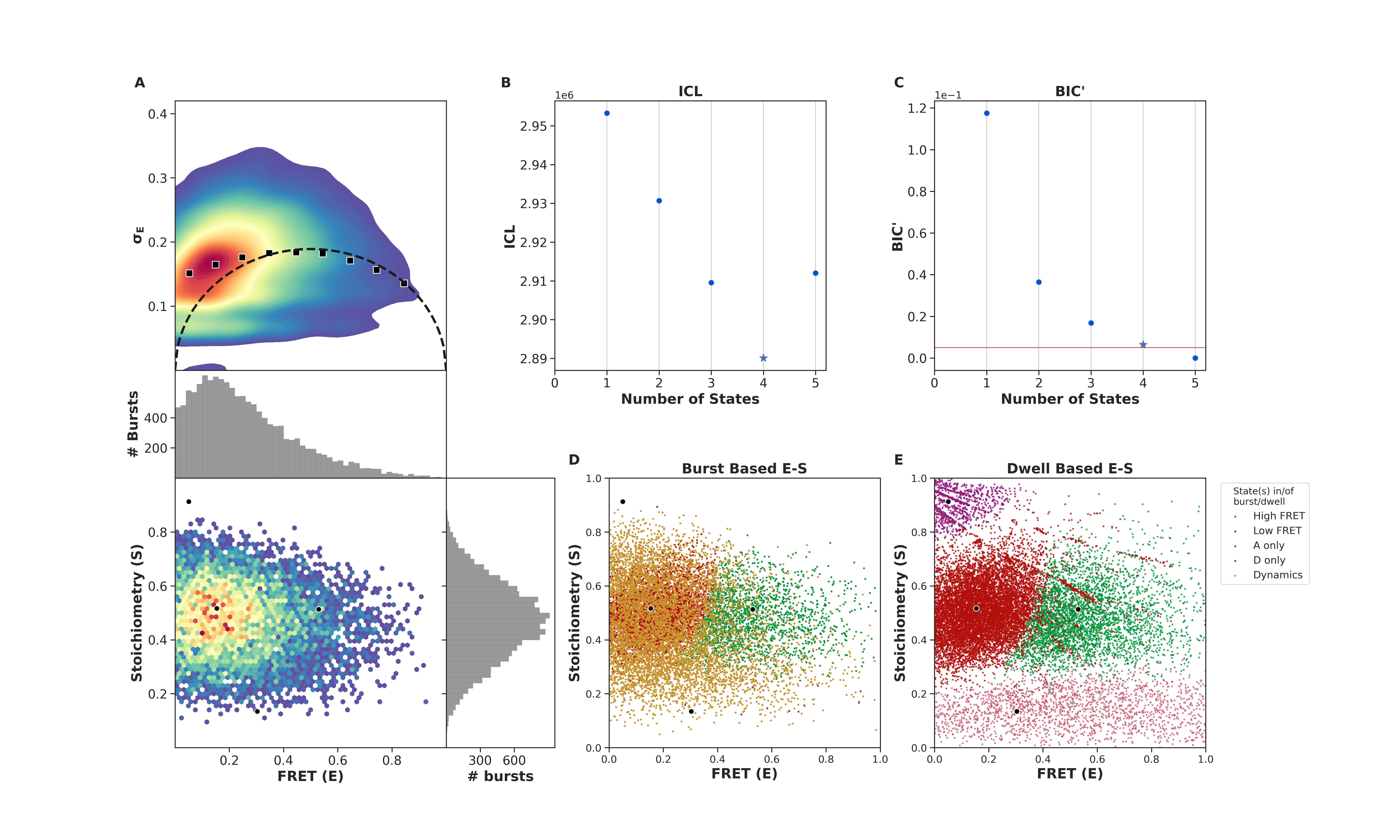

Supplement: Table 2—source data 1. — (A) From top to bottom: (1) Burst variance analysis of the bursts which were corrected by the leakage, crosstalk, and γ-correction factors and which were selected after removing donor-only and acceptor-only bursts. The standard deviation of FRET in each burst is plotted against its mean FRET. Black squares show average values per FRET bin. Black dotted line shows the expected standard deviation in the absence of within-burst dynamics. (2) 2D E-S histogram shows the same data as in (1), with on both sides a histogram that represents the same bursts. (B) Plot of the ICL-values for each final model. The model used in the downstream analysis and following figures is shown as a star. (C) Plot of the BIC’-values for each final model. The red line represents a 0.05 cut-off. The model used is shown as a star. (D) Burst-based 2D E-S scatter plot. Bursts are colored on the basis of the assigned state of the chosen mpH2MM model. If a burst contains more than one state, it is assigned as being dynamic. (E) Dwell-based 2D E-S scatter plot. Dwells are colored on the basis of the assigned state of the chosen mpH2MM model. The dwells were corrected for leakage, direct excitation and the γ-factor. Black dots in A, D and E represent the average value of each state. [file elife-90996-table2-data1.zip › Table 2-Source Data 1/50HEPES600KCl20MgATP100GB500VO4.png]

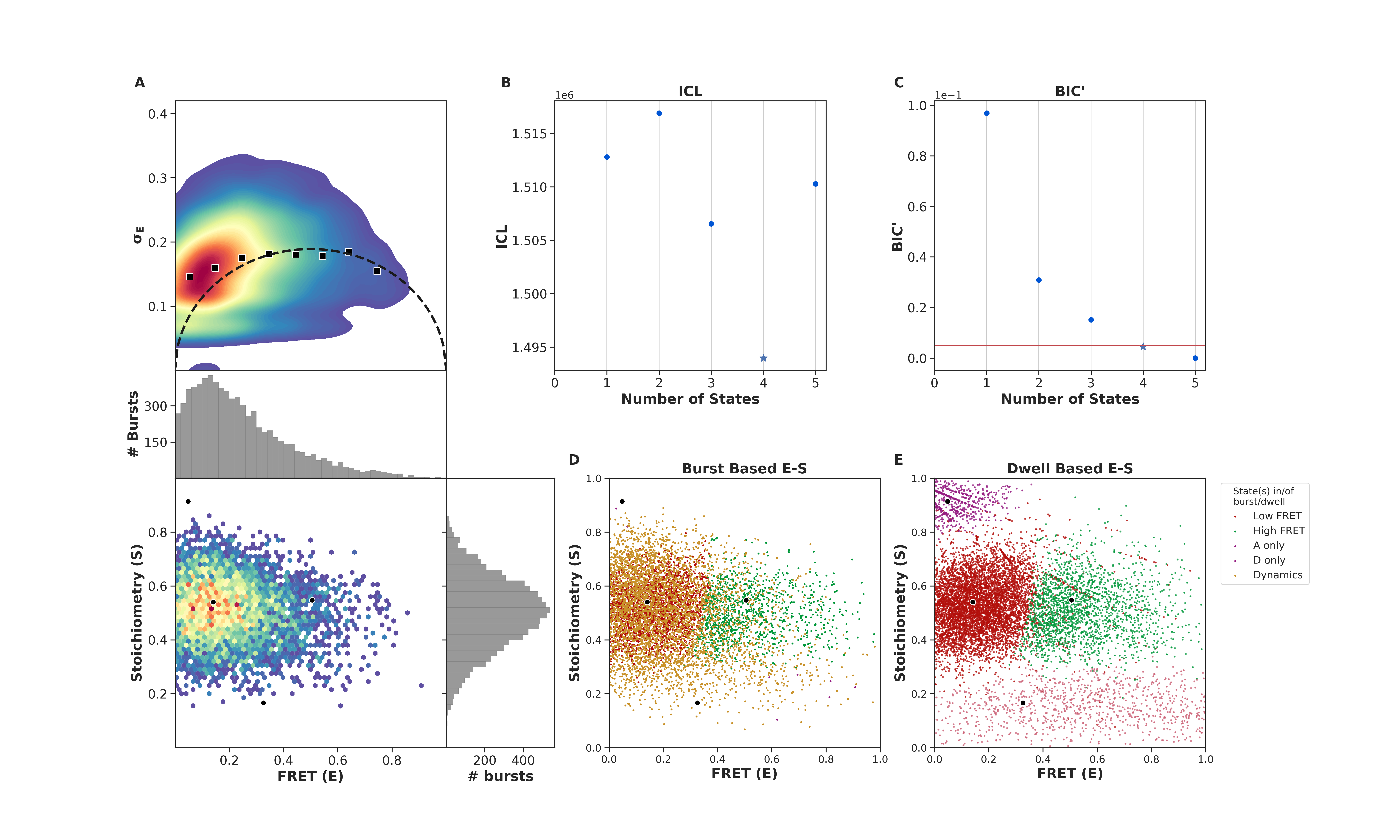

Supplement: Table 2—source data 1. — (A) From top to bottom: (1) Burst variance analysis of the bursts which were corrected by the leakage, crosstalk, and γ-correction factors and which were selected after removing donor-only and acceptor-only bursts. The standard deviation of FRET in each burst is plotted against its mean FRET. Black squares show average values per FRET bin. Black dotted line shows the expected standard deviation in the absence of within-burst dynamics. (2) 2D E-S histogram shows the same data as in (1), with on both sides a histogram that represents the same bursts. (B) Plot of the ICL-values for each final model. The model used in the downstream analysis and following figures is shown as a star. (C) Plot of the BIC’-values for each final model. The red line represents a 0.05 cut-off. The model used is shown as a star. (D) Burst-based 2D E-S scatter plot. Bursts are colored on the basis of the assigned state of the chosen mpH2MM model. If a burst contains more than one state, it is assigned as being dynamic. (E) Dwell-based 2D E-S scatter plot. Dwells are colored on the basis of the assigned state of the chosen mpH2MM model. The dwells were corrected for leakage, direct excitation and the γ-factor. Black dots in A, D and E represent the average value of each state. [file elife-90996-table2-data1.zip › Table 2-Source Data 1/50HEPES600KCl20MgATP100GB-E190Q.png]

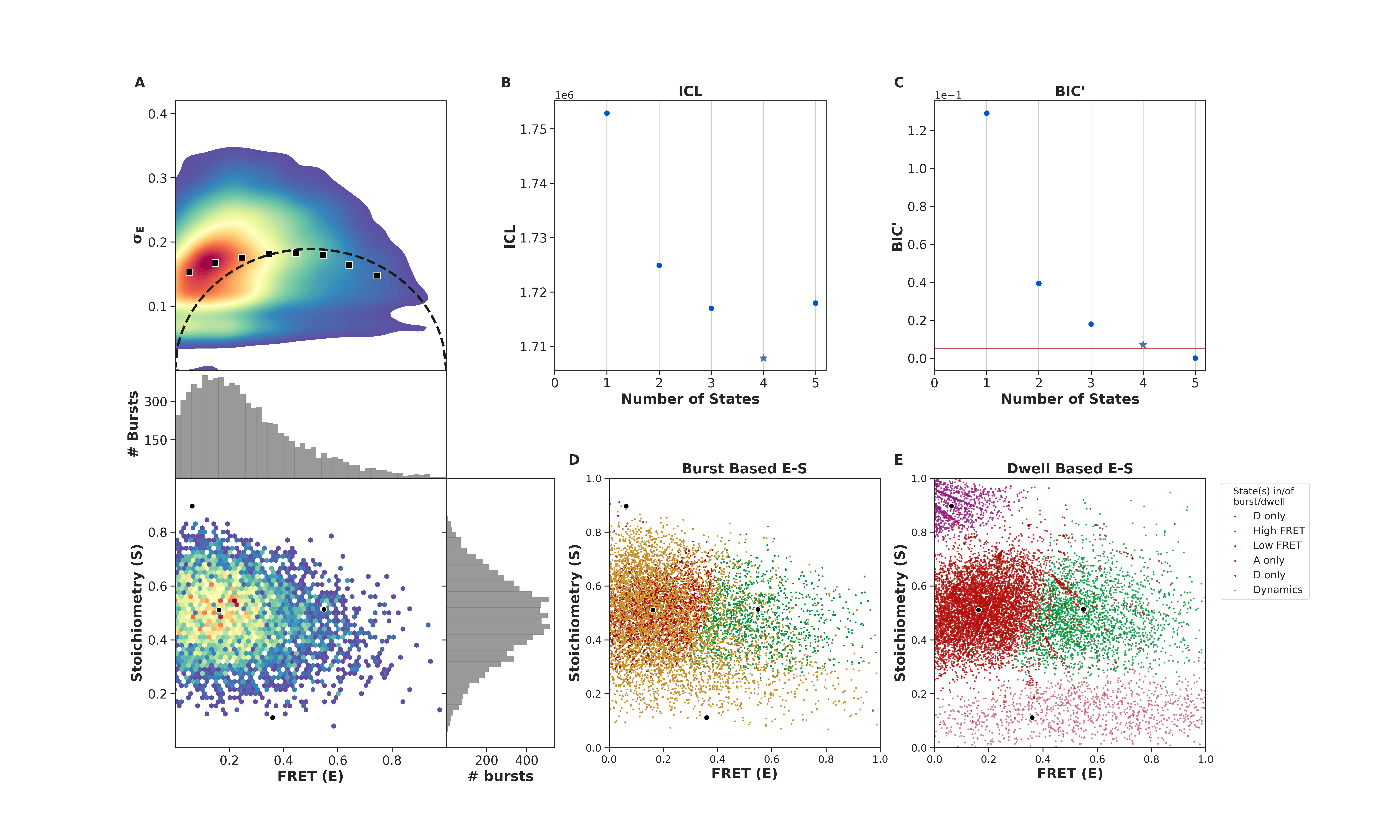

Supplement: Table 2—source data 1. — (A) From top to bottom: (1) Burst variance analysis of the bursts which were corrected by the leakage, crosstalk, and γ-correction factors and which were selected after removing donor-only and acceptor-only bursts. The standard deviation of FRET in each burst is plotted against its mean FRET. Black squares show average values per FRET bin. Black dotted line shows the expected standard deviation in the absence of within-burst dynamics. (2) 2D E-S histogram shows the same data as in (1), with on both sides a histogram that represents the same bursts. (B) Plot of the ICL-values for each final model. The model used in the downstream analysis and following figures is shown as a star. (C) Plot of the BIC’-values for each final model. The red line represents a 0.05 cut-off. The model used is shown as a star. (D) Burst-based 2D E-S scatter plot. Bursts are colored on the basis of the assigned state of the chosen mpH2MM model. If a burst contains more than one state, it is assigned as being dynamic. (E) Dwell-based 2D E-S scatter plot. Dwells are colored on the basis of the assigned state of the chosen mpH2MM model. The dwells were corrected for leakage, direct excitation and the γ-factor. Black dots in A, D and E represent the average value of each state. [file elife-90996-table2-data1.zip › Table 2-Source Data 1/50HEPES600KCl50Glu50Arg.png]

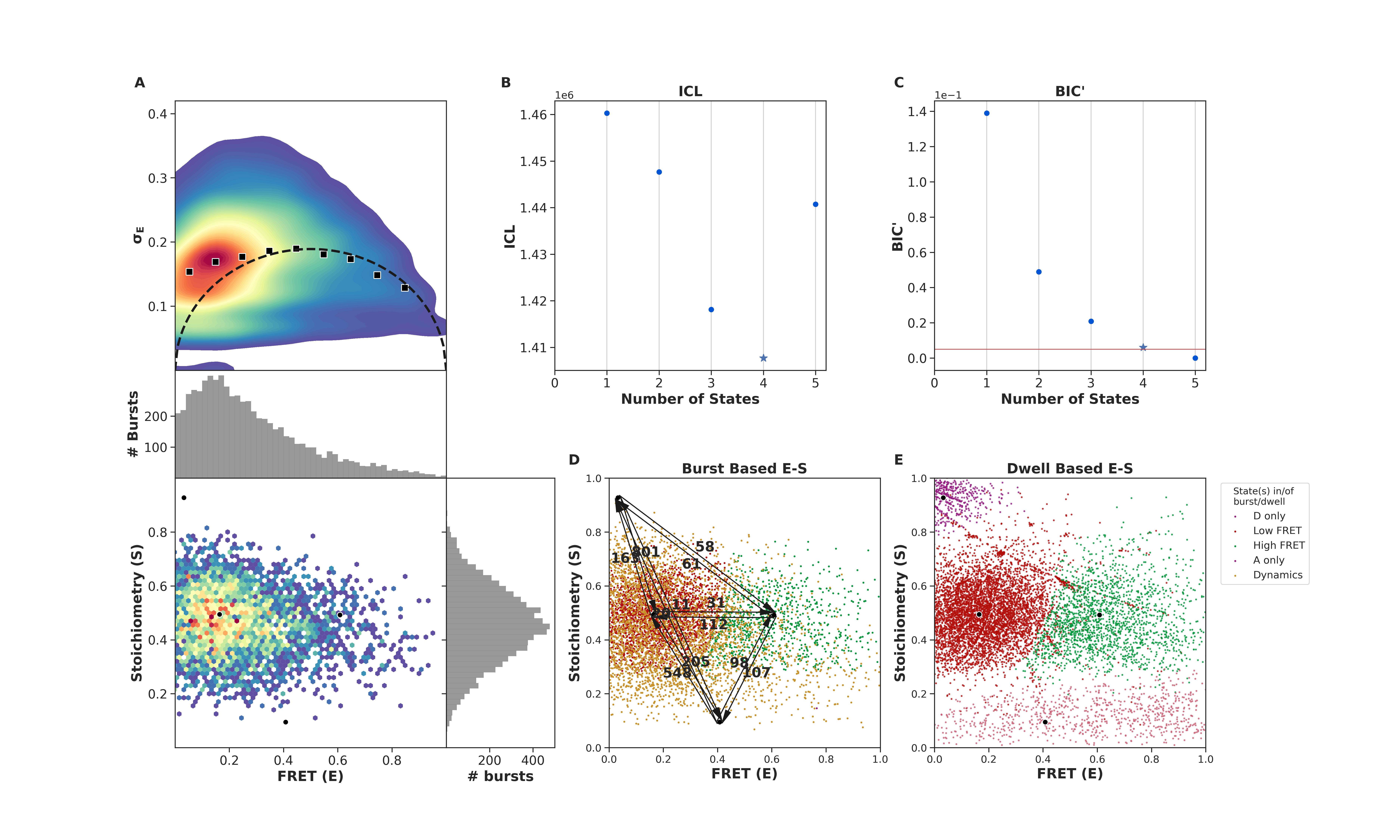

Supplement: Table 2—source data 1. — (A) From top to bottom: (1) Burst variance analysis of the bursts which were corrected by the leakage, crosstalk, and γ-correction factors and which were selected after removing donor-only and acceptor-only bursts. The standard deviation of FRET in each burst is plotted against its mean FRET. Black squares show average values per FRET bin. Black dotted line shows the expected standard deviation in the absence of within-burst dynamics. (2) 2D E-S histogram shows the same data as in (1), with on both sides a histogram that represents the same bursts. (B) Plot of the ICL-values for each final model. The model used in the downstream analysis and following figures is shown as a star. (C) Plot of the BIC’-values for each final model. The red line represents a 0.05 cut-off. The model used is shown as a star. (D) Burst-based 2D E-S scatter plot. Bursts are colored on the basis of the assigned state of the chosen mpH2MM model. If a burst contains more than one state, it is assigned as being dynamic. (E) Dwell-based 2D E-S scatter plot. Dwells are colored on the basis of the assigned state of the chosen mpH2MM model. The dwells were corrected for leakage, direct excitation and the γ-factor. Black dots in A, D and E represent the average value of each state. [file elife-90996-table2-data1.zip › Table 2-Source Data 1/50HEPES600KCl-V149Q.png]

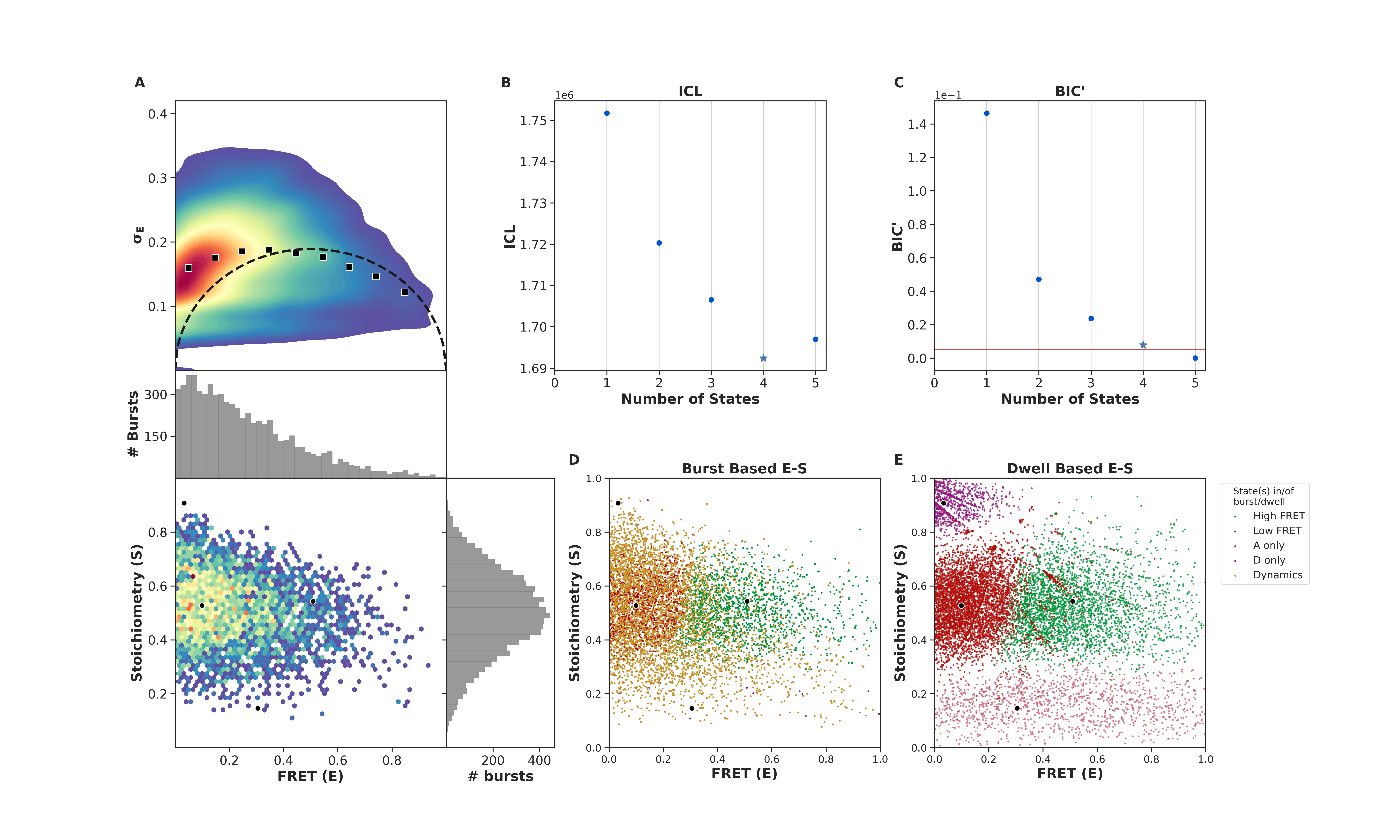

Supplement: Table 3—source data 1. — (A) From top to bottom: (1) Burst variance analysis of the bursts which were corrected by the leakage, crosstalk, and γ-correction factors and which were selected after removing donor-only and acceptor-only bursts. The standard deviation of FRET in each burst is plotted against its mean FRET. Black squares show average values per FRET bin. Black dotted line shows the expected standard deviation in the absence of within-burst dynamics. (2) 2D E-S histogram shows the same data as in (1), with on both sides a histogram that represents the same bursts. (B) Plot of the ICL-values for each final model. The model used in the downstream analysis and following figures is shown as a star. (C) Plot of the BIC’-values for each final model. The red line represents a 0.05 cut-off. The model used is shown as a star. (D) Burst-based 2D E-S scatter plot. Bursts are colored on the basis of the assigned state of the chosen mpH2MM model. If a burst contains more than one state, it is assigned as being dynamic. (E) Dwell-based 2D E-S scatter plot. Dwells are colored on the basis of the assigned state of the chosen mpH2MM model. The dwells were corrected for leakage, direct excitation and the γ-factor. Black dots in A, D and E represent the average value of each state. [file elife-90996-table3-data1.zip › Table 3-Source Data 1/50HEPES0KCl.png]

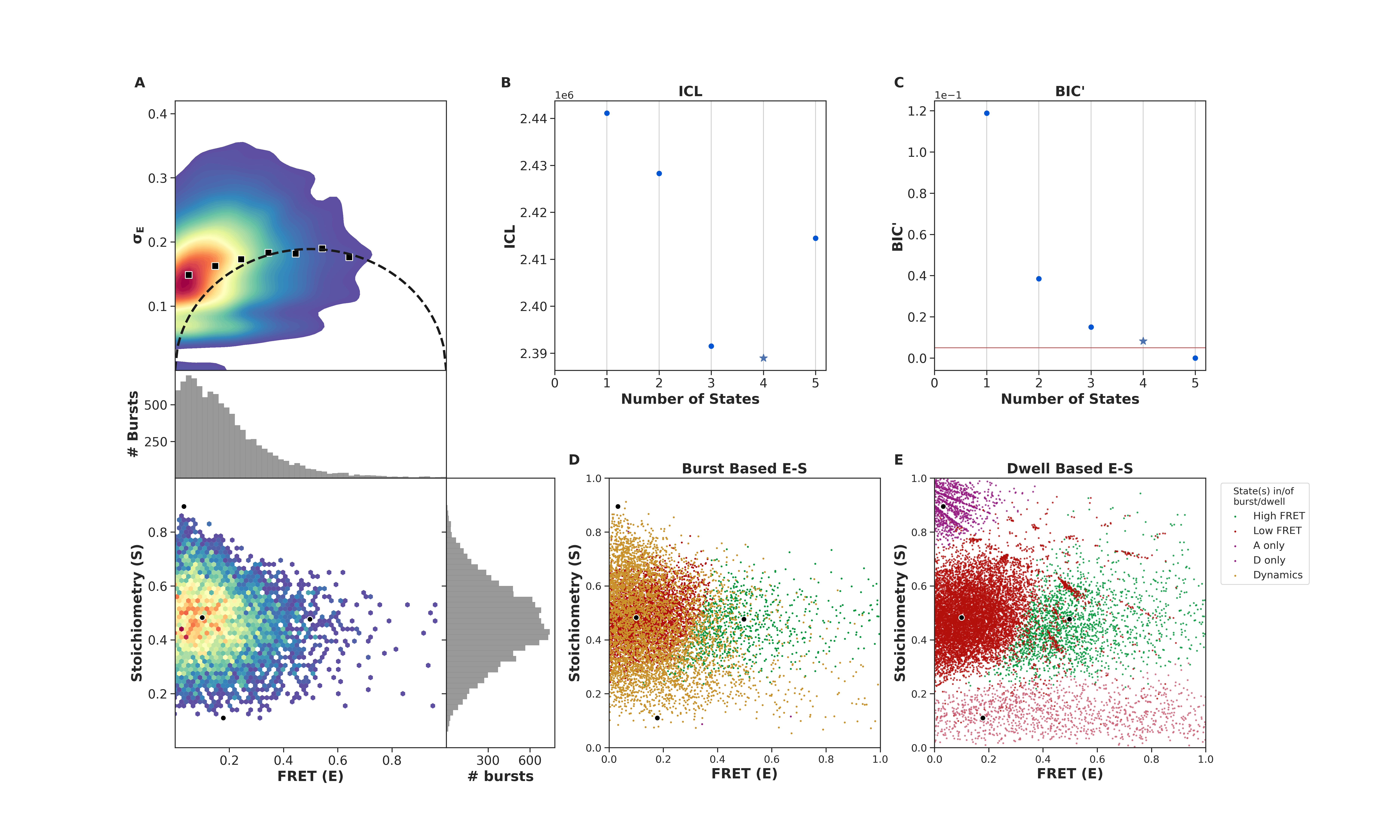

Supplement: Table 3—source data 1. — (A) From top to bottom: (1) Burst variance analysis of the bursts which were corrected by the leakage, crosstalk, and γ-correction factors and which were selected after removing donor-only and acceptor-only bursts. The standard deviation of FRET in each burst is plotted against its mean FRET. Black squares show average values per FRET bin. Black dotted line shows the expected standard deviation in the absence of within-burst dynamics. (2) 2D E-S histogram shows the same data as in (1), with on both sides a histogram that represents the same bursts. (B) Plot of the ICL-values for each final model. The model used in the downstream analysis and following figures is shown as a star. (C) Plot of the BIC’-values for each final model. The red line represents a 0.05 cut-off. The model used is shown as a star. (D) Burst-based 2D E-S scatter plot. Bursts are colored on the basis of the assigned state of the chosen mpH2MM model. If a burst contains more than one state, it is assigned as being dynamic. (E) Dwell-based 2D E-S scatter plot. Dwells are colored on the basis of the assigned state of the chosen mpH2MM model. The dwells were corrected for leakage, direct excitation and the γ-factor. Black dots in A, D and E represent the average value of each state. [file elife-90996-table3-data1.zip › Table 3-Source Data 1/50HEPES600KCl.png]

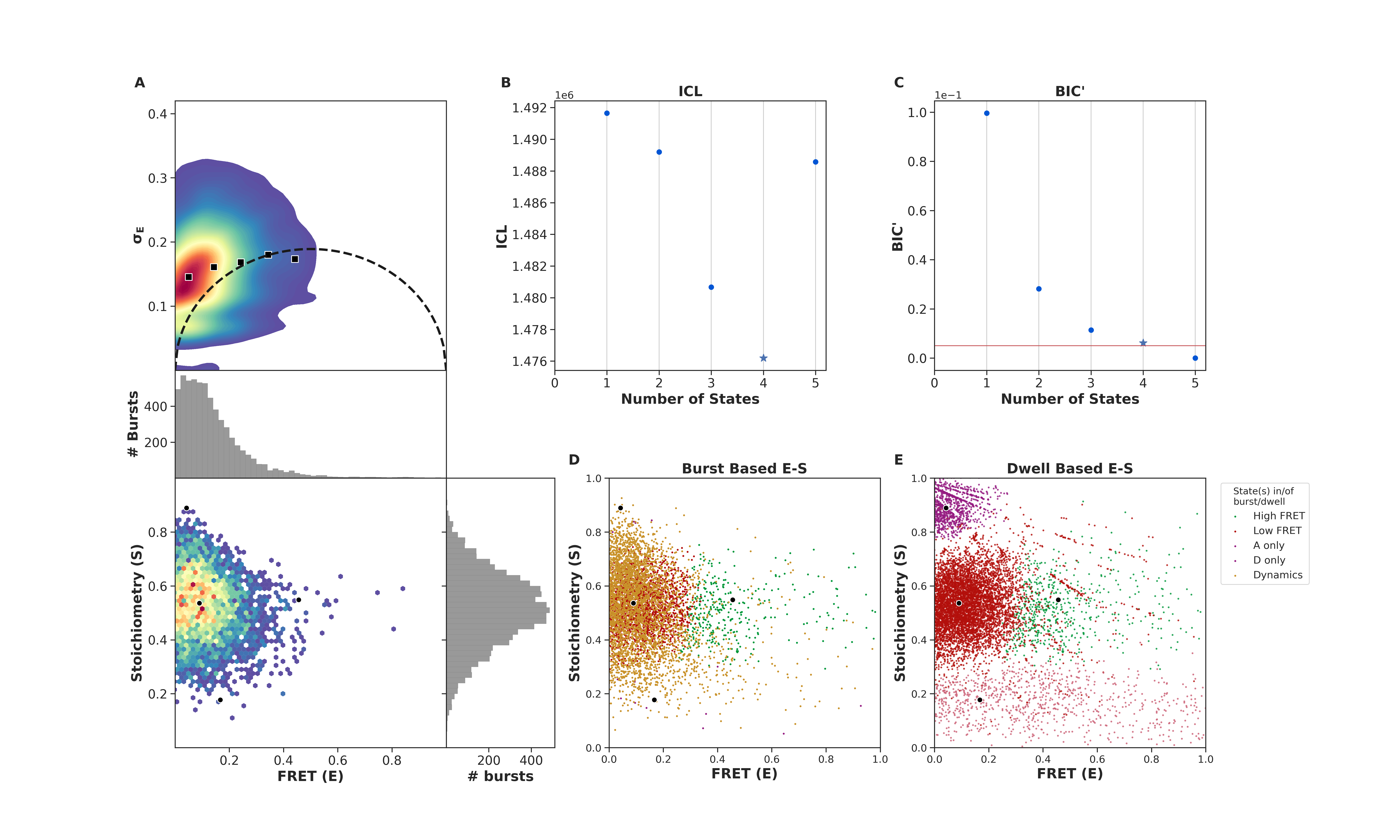

Supplement: Table 3—source data 1. — (A) From top to bottom: (1) Burst variance analysis of the bursts which were corrected by the leakage, crosstalk, and γ-correction factors and which were selected after removing donor-only and acceptor-only bursts. The standard deviation of FRET in each burst is plotted against its mean FRET. Black squares show average values per FRET bin. Black dotted line shows the expected standard deviation in the absence of within-burst dynamics. (2) 2D E-S histogram shows the same data as in (1), with on both sides a histogram that represents the same bursts. (B) Plot of the ICL-values for each final model. The model used in the downstream analysis and following figures is shown as a star. (C) Plot of the BIC’-values for each final model. The red line represents a 0.05 cut-off. The model used is shown as a star. (D) Burst-based 2D E-S scatter plot. Bursts are colored on the basis of the assigned state of the chosen mpH2MM model. If a burst contains more than one state, it is assigned as being dynamic. (E) Dwell-based 2D E-S scatter plot. Dwells are colored on the basis of the assigned state of the chosen mpH2MM model. The dwells were corrected for leakage, direct excitation and the γ-factor. Black dots in A, D and E represent the average value of each state. [file elife-90996-table3-data1.zip › Table 3-Source Data 1/50HEPES600KCl20ATP.png]

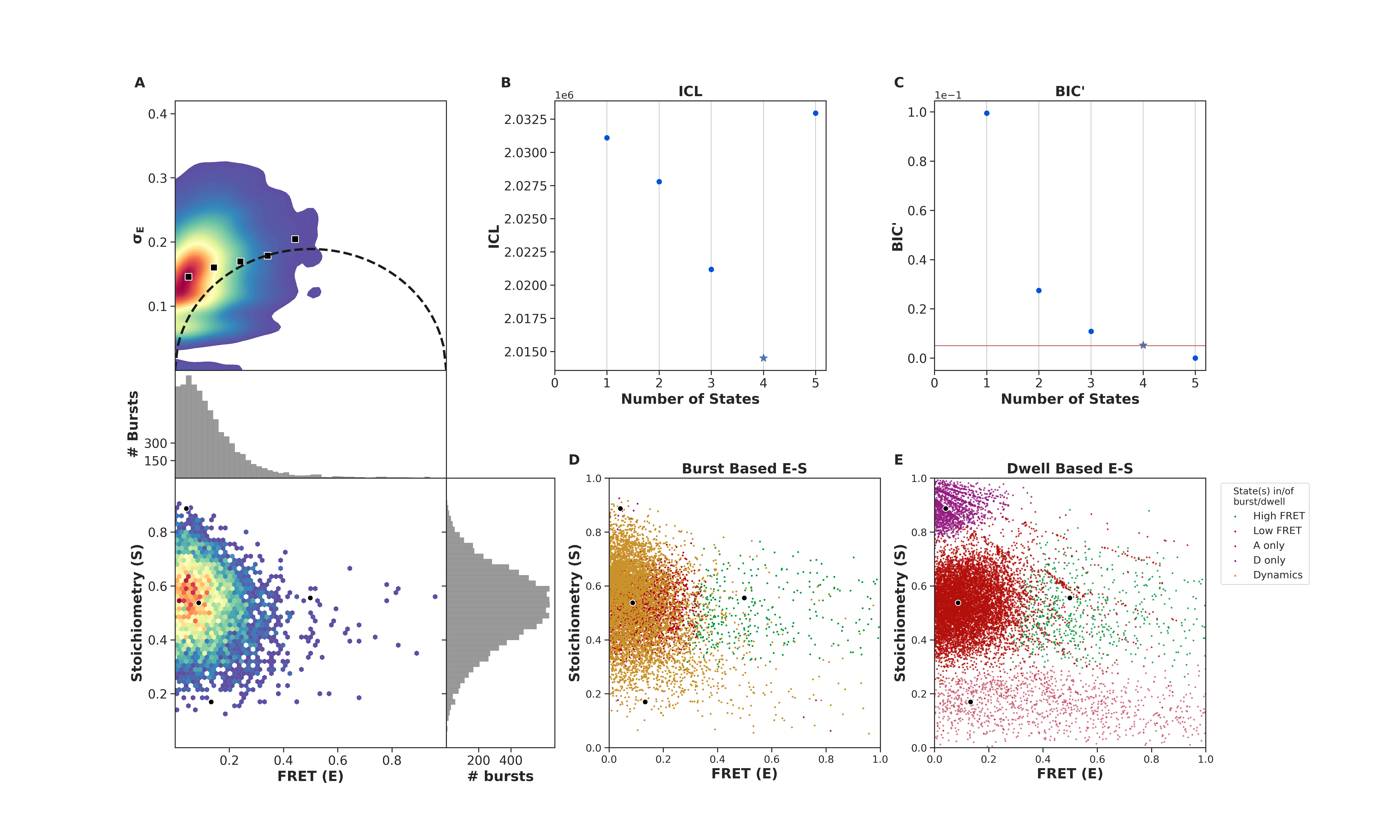

Supplement: Table 3—source data 1. — (A) From top to bottom: (1) Burst variance analysis of the bursts which were corrected by the leakage, crosstalk, and γ-correction factors and which were selected after removing donor-only and acceptor-only bursts. The standard deviation of FRET in each burst is plotted against its mean FRET. Black squares show average values per FRET bin. Black dotted line shows the expected standard deviation in the absence of within-burst dynamics. (2) 2D E-S histogram shows the same data as in (1), with on both sides a histogram that represents the same bursts. (B) Plot of the ICL-values for each final model. The model used in the downstream analysis and following figures is shown as a star. (C) Plot of the BIC’-values for each final model. The red line represents a 0.05 cut-off. The model used is shown as a star. (D) Burst-based 2D E-S scatter plot. Bursts are colored on the basis of the assigned state of the chosen mpH2MM model. If a burst contains more than one state, it is assigned as being dynamic. (E) Dwell-based 2D E-S scatter plot. Dwells are colored on the basis of the assigned state of the chosen mpH2MM model. The dwells were corrected for leakage, direct excitation and the γ-factor. Black dots in A, D and E represent the average value of each state. [file elife-90996-table3-data1.zip › Table 3-Source Data 1/50HEPES600KCl20ATP100GB.png]

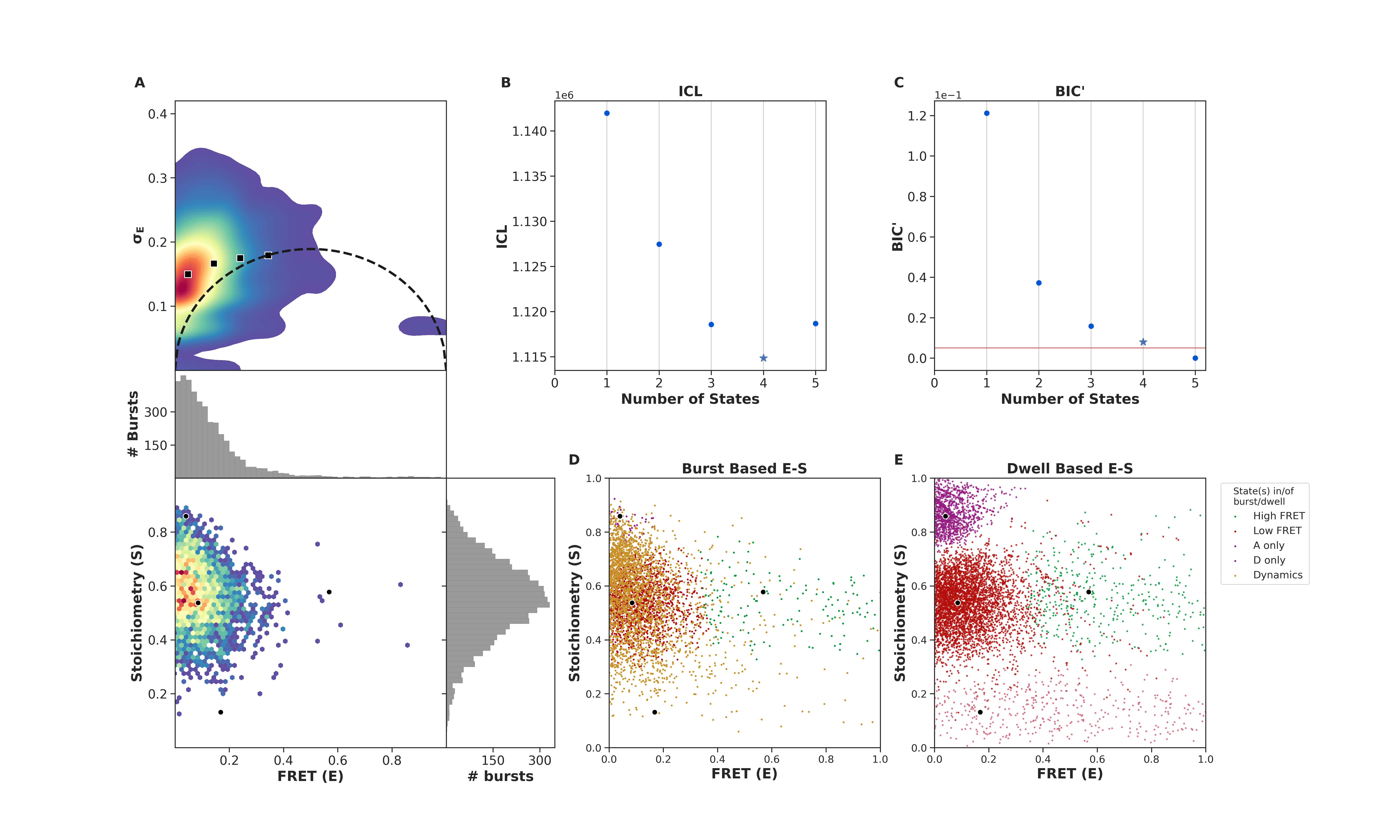

Supplement: Table 3—source data 1. — (A) From top to bottom: (1) Burst variance analysis of the bursts which were corrected by the leakage, crosstalk, and γ-correction factors and which were selected after removing donor-only and acceptor-only bursts. The standard deviation of FRET in each burst is plotted against its mean FRET. Black squares show average values per FRET bin. Black dotted line shows the expected standard deviation in the absence of within-burst dynamics. (2) 2D E-S histogram shows the same data as in (1), with on both sides a histogram that represents the same bursts. (B) Plot of the ICL-values for each final model. The model used in the downstream analysis and following figures is shown as a star. (C) Plot of the BIC’-values for each final model. The red line represents a 0.05 cut-off. The model used is shown as a star. (D) Burst-based 2D E-S scatter plot. Bursts are colored on the basis of the assigned state of the chosen mpH2MM model. If a burst contains more than one state, it is assigned as being dynamic. (E) Dwell-based 2D E-S scatter plot. Dwells are colored on the basis of the assigned state of the chosen mpH2MM model. The dwells were corrected for leakage, direct excitation and the γ-factor. Black dots in A, D and E represent the average value of each state. [file elife-90996-table3-data1.zip › Table 3-Source Data 1/50HEPES600KCl20ATP100GB500VO4.png]

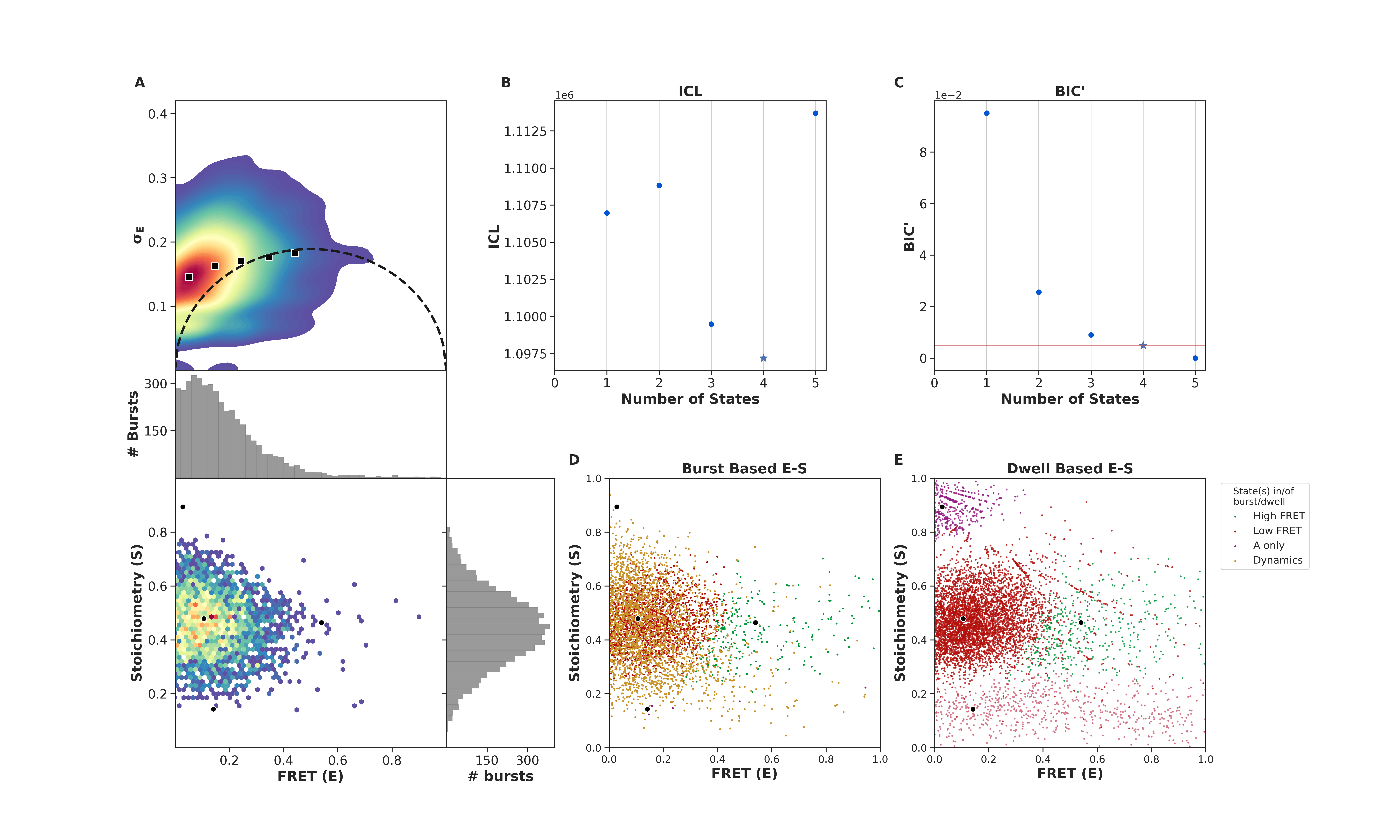

Supplement: Table 3—source data 1. — (A) From top to bottom: (1) Burst variance analysis of the bursts which were corrected by the leakage, crosstalk, and γ-correction factors and which were selected after removing donor-only and acceptor-only bursts. The standard deviation of FRET in each burst is plotted against its mean FRET. Black squares show average values per FRET bin. Black dotted line shows the expected standard deviation in the absence of within-burst dynamics. (2) 2D E-S histogram shows the same data as in (1), with on both sides a histogram that represents the same bursts. (B) Plot of the ICL-values for each final model. The model used in the downstream analysis and following figures is shown as a star. (C) Plot of the BIC’-values for each final model. The red line represents a 0.05 cut-off. The model used is shown as a star. (D) Burst-based 2D E-S scatter plot. Bursts are colored on the basis of the assigned state of the chosen mpH2MM model. If a burst contains more than one state, it is assigned as being dynamic. (E) Dwell-based 2D E-S scatter plot. Dwells are colored on the basis of the assigned state of the chosen mpH2MM model. The dwells were corrected for leakage, direct excitation and the γ-factor. Black dots in A, D and E represent the average value of each state. [file elife-90996-table3-data1.zip › Table 3-Source Data 1/50HEPES600KCl20MgATP100GB-E190Q.png]
